# Topological constraints of RNA pseudoknotted and loop-kissing motifs: applications to three-dimensional structure prediction

**DOI:** 10.1093/nar/gkaa463

**Published:** 2020-06-03

**Authors:** Xiaojun Xu, Shi-Jie Chen

**Affiliations:** Institute of Bioinformatics and Medical Engineering, Jiangsu University of Technology, Changzhou, Jiangsu 213001, China; Department of Physics, Department of Biochemistry, and Informatics Institute, University of Missouri, Columbia, MO 65211, USA

## Abstract

An RNA global fold can be described at the level of helix orientations and relatively flexible loop conformations that connect the helices. The linkage between the helices plays an essential role in determining the structural topology, which restricts RNA local and global folds, especially for RNA tertiary structures involving cross-linked base pairs. We quantitatively analyze the topological constraints on RNA 3D conformational space, in particular, on the distribution of helix orientations, for pseudoknots and loop-loop kissing structures. The result shows that a viable conformational space is predominantly determined by the motif type, helix size, and loop size, indicating a strong topological coupling between helices and loops in RNA tertiary motifs. Moreover, the analysis indicates that (cross-linked) tertiary contacts can cause much stronger topological constraints on RNA global fold than non-cross-linked base pairs. Furthermore, based on the topological constraints encoded in the 2D structure and the 3D templates, we develop a 3D structure prediction approach. This approach can be further combined with structure probing methods to expand the capability of computational prediction for large RNA folds.

## INTRODUCTION

Most RNAs fold in a hierarchical pathway, with the folding of secondary structures typically preceding the formation of tertiary interactions (1–3). Chain (bond) connectivity, excluded volume, and the linkage between the different helices, play an essential role in shaping the RNA conformational space and the global topology of the native structure (4–16). Different RNA 2D structural motifs show different linkages between helices. Understanding the degree to which RNA structural topology restricts RNA 3D conformational space (i.e. topological constraints) can improve the accuracy of structure prediction as well as our understanding for RNA folding principles.

Compared with the protein backbone, RNA contains more rotatable bonds per residue, contributing to the substantial conformational flexibility. Muthy et al. performed a grid search for all the potential conformers for a dinucleotide, and found that hard sphere steric exclusion and bond connectivity can restrict torsion angles in nucleic acids to <5% of all the possible conformations (4). For the helix-junction-helix (HJH) motif, Chu et. al. studied two helices joined by flexible single- or double-stranded polyethylene glycol tethers (5). Stochastic dynamics simulations and small-angle X-ray scattering experiments showed that the HJH junction topology can further significantly reduce the conformational space and influence the preferred location and orientation of the adjoining helices. Conformational analysis based on TOPRNA (6) and MC-sym (7,8) showed that RNA global conformation is largely defined by topological constraints of RNA secondary structure while the electrostatics, intra- and inter-loop and other interactions select specific conformations from the accessible conformational ensemble.

Different approaches have been developed to investigate the impact of RNA 2D structural constraint on 3D conformations. For example, guided by the knowledge-based statistical potentials for bending and torsional degrees of freedom of the internal loop and radius of gyration, graph theory-based tool (RAG) (14,15) can efficiently sample the global helical topologies (as represented by graphs) in 3D space. Ernwin (16), a coarse-grained helix-centered model as another example, explores global arrangements of helices and loops within RNA structures. Combined with a novel energy function for the positioning of stems and loops, the model can predict RNA 3D structures.

RNA 3D structure prediction (17–39), on the other hand, has been developed for decades to help in understanding RNA structure-function relationships, as well as to promote the rational design of RNA-based molecular systems. The prediction methods result from various coarse-grained levels (23–30), such as iFoldRNA (27), SimRNA (28,29), and IsRNA (30), have the ability to predict RNA structures and folding kinetics, but the accuracy of the predictions can be limited by the large conformational space for some RNAs. Template-assembly approaches build RNA 3D structures based on the known structural modules ranging from piece-wise fragments to whole structural motifs (8,31–39). For example, FARNA/FARFAR (34,35) predicts RNA structures through the assembly of 1–3 nucleotides from known structures via a Monte Carlo procedure. It can reach atomic resolution for most short RNAs (<30 nt). MC-sym (8), RNAcomposer (36) and Vfold3D (37,38), furthermore, take the advantage of strong topological constraints of secondary structural (bulge, internal loops and multi-branched junctions, shown in Supplementary Figure S1) motifs, and select templates from known structures with homologous sequence information to predict RNA 3D structures.

RNA structures often involve cross-linked tertiary base pairs (as indicated in the circular representations in Supplementary Figure S1), such as pseudoknots (PK) and hairpin-hairpin loop kissing (KISS) motifs. Most motif template-based RNA structure prediction methods use the motif type and loop/helix size-dependent scoring scheme to search for appropriate templates to build structures. However, the success rate to find a proper template is usually low for large RNAs with tertiary contacts. A possible approach to alleviate the problem of limited availability of templates is to search for the topology-based templates, i.e. templates with the same motif type but may have different sizes and sequences for the loops and helices. Here, we present a comprehensive analysis for the topological constraints of four types of RNA structural motifs with tertiary (cross-linked) contacts. Specifically, we investigate the helix and loop size dependence of the conformational space, particularly the topological constraints induced by the cross-linked base pairs in the PK and KISS motifs. We use the fractional change in the number of viable conformations to quantify the topological constraint. Compared with the RNA motifs without cross-linked base pairs, the tertiary contacts impose much stronger topological constraints on RNA global folds. Therefore, the use of cross-linked contacts can significantly improve the conformational sampling quality for 3D structure prediction. Furthermore, we here also develop and illustrate a multi-stage method, which incorporates Vfold3D, VfoldLA (39) and tertiary topologies, in RNA 3D structure prediction. By taking the advantage of the strong topological constraints imposed by the tertiary contacts, as shown in the benchmark tests, our multi-stage model can provide improved predictions for RNA 3D structures with cross-linked base pairs.

## ALGORITHM AND METHODS

### Transformation matrix for helix orientation

**Figure 1.**
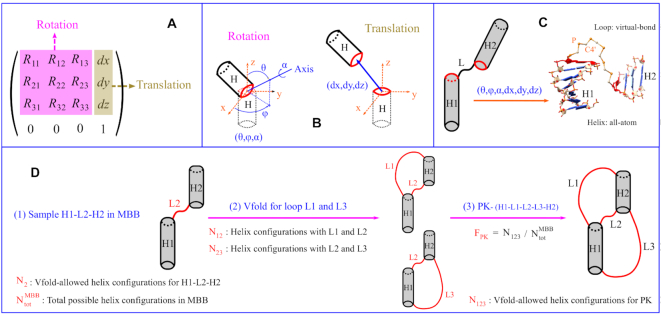
(**A**) Transformation matrix for the rigid-body translational and rotational transformations. (**B**) The nine-element rotation matrix in (A) is characterized by three independent parameters for the rotation axis (θ, φ) passing through the geometric center of the first base pair (red oval), with the rotation angle α. Vector (*dx*, *dy*, *dz*) denotes the translational displacement between the helices. (**C**) The definition of an HLH motif, and the sampling of the 3D helix configurations defined by (θ, φ, α, *dx*, *dy*, *dz*) with Vfold. Helices are modeled with all atom A-form structures. Loops are generated by the Vfold (P-C4′-P) backbone structures. (**D**) Topological constraints for PK, which contains two helices and three loops. The HLH of H1–L2–H2 is used to sample the helix configurations in MBB. Loops are modeled by Vfold to determine the allowed and disallowed helix configurations. *N*_x_ is the number of allowed helix configurations with the respective loop connections. *F*_PK_ is the fraction of Vfold-allowed helix configurations of a PK motif.

The different configurations for a pair of helices can be generated through the translation-rotation transformation between the helices. Mathematically, the transformation of a rigid body can be described by a 4 × 4 matrix for the translation and rotation, as shown in Figure 1(A). Vector (*dx*, *dy*, *dz*) denotes the translational displacement between the helices, and the nine-element 3 × 3 submatrix **R** represents the rotation with three independent parameters: polar angle θ, azimuthal angle φ for the rotation axis, and rotation angle α, as shown in Figure 1(B). Therefore, the spatial configuration of a two-helix system can be described and generated by six parameters (θ, φ, α, *dx*, *dy*, *dz*). Correspondingly, the parameters for any given helix configuration can be uniquely extracted from the relative displacement and orientation between the first base pairs of the helices (see the red oval in Figure 1B).

### Vfold for allowed/disallowed configurations

To quantify the degree to which structural topology restricts the sampling of 3D conformations, we use the coarse-grained representation of RNA structures (Vfold model) (40) and sample the coarse-grained loop structures with given motif type, helix sizes and loop sizes.

- For a given helix–loop–helix (HLH) 2D motif, to determine the minimal bounding box (MBB) for the 3D conformations, we use Vfold to sample the coarse-grained 3D conformations; See the P–C4’–P virtual-bond loop structures in Figure 1C. We choose the geometric center of the terminal base pair in one of the helices as the center of the bounding box. In the conformational sampling, the (geometric center of) terminal base pair in the other helix sweeps out a 3D region. The minimum box that contains such a region defines the MBB. See the Supplementary Figure S2 for detailed descriptions about the Vfold model and the MBB calculations. By definition of the MBB, we only need to sample HLH configurations within the minimal bounding box.
  In the MBB calculations shown in the Supplementary Figure S2, we sample the orientation of the rotation axis (θ, φ) with 100 quasi-uniformly distributed points on the surface of a sphere (see Supplementary Figure S3). We use 18 uniformly spaced rotation angles α between 0 and 2π to rotate the helices for each given rotation axis, which leads to *R* = 1800 total rotations. To achieve the optimal balance between the computational efficiency and the sampling quality, we use loop-size dependent adaptive grid size ((*L* + 1) Å for an L-nt loop) in MBB for translational displacement of rotated helices. Specifically, the total grids in MBB of HLH with *L* = 0, 1, 2, and 3 nt are *T* = 23548, 8820, 4913 and 3840, respectively. The total possible helical orientations is equal to *N*$_{\rm tot}^{\rm MBB}$ = *R* × *T*. For example, *N*$_{\rm tot}^{\rm MBB}$ = 1800 × 8820 for an HLH motif with *L* = 1 nt (grid size of 2 Å in MBB).
- We generate the possible helix configurations based on (θ, φ, α, *dx*, *dy*, *dz*). For the PK motif with two helices and three loops, denoted by the size of H1–L1–L2–L3–H2 (see Supplementary Figure S4), we use the HLH motif of H1–L2–H2 to exhaustively sample the Vfold-allowed two-helix configurations within its MBB. *N*$_{\rm tot}^{\rm MBB}$ of H1–L2–H2 gives the total number of the possible two-helix configurations described by (θ, φ, α, *dx*, *dy*, *dz*) for a PK motif.
  The KISS motif, as illustrated in Supplementary Figure S4, contains three helices and six loops, denoted by the size of (H1–H2–H3)-(L1–L2–L3–L4–L5–L6). In order to sample the conformational ensemble with viable computational feasibility, we randomly sample the three-helix configurations with the HLHs of H1–L2–H2, and H2–L4–H3 in their corresponding MBBs. As shown in Supplementary Figure S5, *N*$_{\rm tot}^{\rm 24}$ gives the total randomly sampled (three-helix) helical configurations of a KISS motif, characterized by two sets of (θ, φ, α, *dx*, *dy*, *dz*).
- For each given helix configuration, we generate Vfold loop conformations with the consideration of helix-loop excluded volume interactions. If at least one viable conformation can be realized for the loops, the given helix configuration is considered as being acceptable (allowed), otherwise, it is considered topologically unacceptable (disallowed). As shown in Figure 1D and Supplementary Figure S5, we take count of the allowed helix configurations for the two-helix (PK) and three-helix (KISS) systems with the different loop linkages. Our purpose is to assess the impact of topological constraints. We use *N*_*x*_ to denote the number of the Vfold-allowed helical configurations of the given helix system. For example, *N*_124_ in Supplementary Figure S5 gives the number of the viable helical configurations of the three helices connected by the three viable loops of L1, L2 and L4. Therefore, the decrease of the number of the viable helical configurations from N_124_ to N_1245_ indicates the impact of the topological constraints from loop L5 in the presence of loops L1, L2 and L4.
- To calculate the fraction of forming viable helical configurations. From the ratio between the numbers of the allowed and the total possible helical conformations, we evaluate the degree to which RNA structural topology restricts the conformations for the different motif types, helix sizes and loop sizes. We use the (topological constraint) fraction *F*_*x*_ = *N*_*x*_/*N*_tot_ (*N*$_{\rm tot}^{\rm MBB}$ for PK and *N*$_{\rm tot}^{\rm 24}$ for KISS) to measure the topological constraints of the helix system with the respective loop connections. Therefore, as shown in Figure 1D and Supplementary Figure S5, *F*_PK_ (= *N*_123_/*N*$_{\rm tot}^{\rm MBB}$) and *F*_KISS_ (= *N*_123456_/*N*$_{\rm tot}^{\rm 24}$) gives the topological constraints of PK and KISS motifs, respectively.

### Topology-based 3D structure prediction

**Figure 2.**
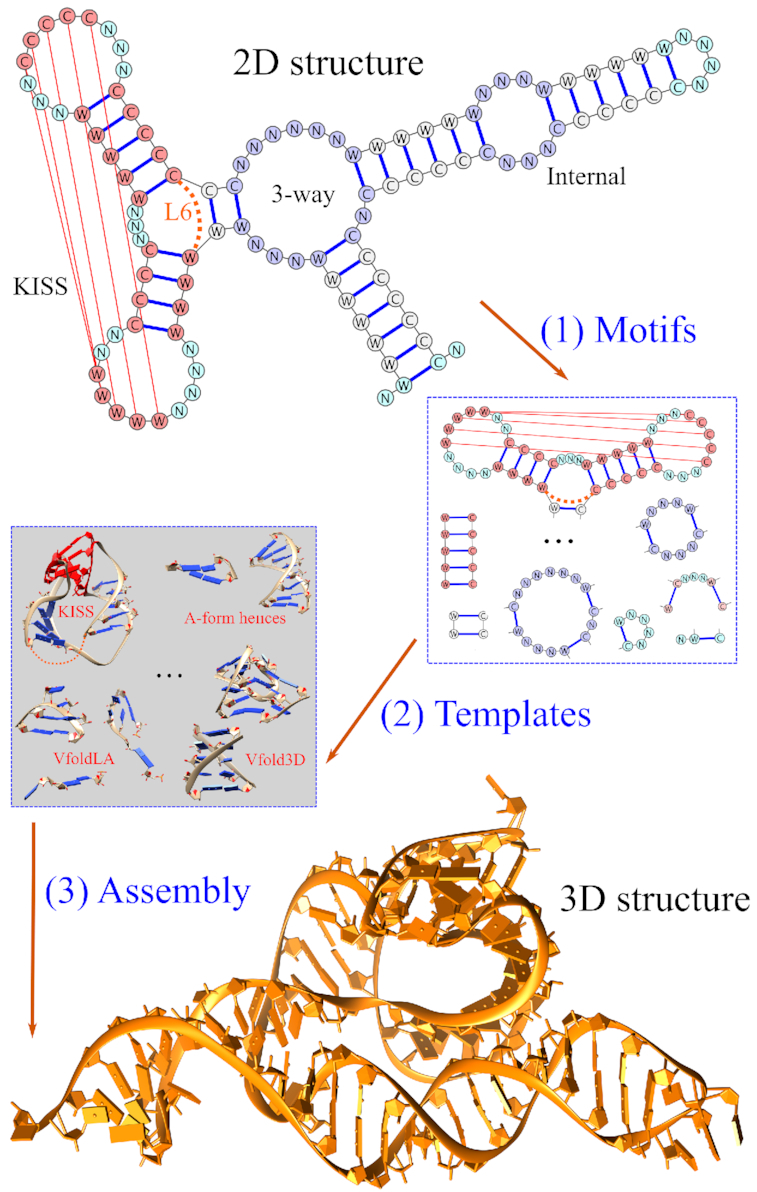
The multi-stage approach, which incorporates Vfold3D, VfoldLA and tertiary topologies, for RNA 3D structure prediction. The method involves three steps: (1) extract motifs from a given 2D structure; (2) select templates from known RNA structures; (3) generate all-atom models to predict the 3D structures.

To take the advantage of structural topological constraints, in particular, from tertiary contacts, we develop a multi-stage RNA 3D structure prediction method. Specifically, as shown in Figure 2, we integrate Vfold3D, VfoldLA and tertiary topologies to predict RNA structures containing cross-linked base pairs.

- To classify motifs with given 2D structure. As shown in Supplementary Figure S1, we define three types of motifs: (a) Tertiary structural motifs, such as PK, and KISS; (b) Secondary structural motifs, such as junctions, internal and bulge loops; (c) Single-stranded loops, such as hairpin, helix2 and tails. For the purpose to determine the irreducible tertiary ‘topology’ of a complicated structure, we replace the isolated substructures, such as helices, stem-loop, and junctions, with the terminal base pairs of the substructures. For example, the given 2D structure in Figure 2 is broken down into the structural motifs of one KISS, one three-way junction, one internal loop, and several helices and single-stranded loops. The size of the KISS motif is (4–5–5)-(4–2–3–3–3–2), with the size of 2-nt (one terminal base pair) for loop L6.
- To build helices with all-atom A-form helical structures.
- To construct 3D structures for tertiary structural motifs. We select the optimal templates from the structural database for the same motif type. It should be noted that the current template database for the PK and KISS motifs is quite limited, compared with the database of the secondary structural motifs. If no templates with the exact helix and loop sizes are found, We choose the templates with the similar helix and loop sizes. We select the optimal templates according to the size-dependent scoring scheme, *S*^PK/KISS^ = *a*∑_helix_Δ*H* + *b*∑_loop_ Δ*L*, where Δ*H* and Δ*L* are the size differences between the corresponding helix and loop, respectively. Here the size of a helix is defined as the number of base pairs. We set the prefactor *a* = 2 for the (helix) Δ*H*-contribution. Since the loops L2 for PK and L2 and L4 for KISS are usually shorter (<4 nts) with stronger topological constraints than other loops. We set *b* = 5 for these loops and 1 for others. For example, the KISS motif extracted from the given 2D structure in Figure 2 is (4–5–5)-(4–2–3–3–3–2). The score for the query template with the size of (5–5–4)-(3–1–4–4–5–2) is 18.
- To build all-atom structures for secondary structural motifs using Vfold3D (37,38). Vfold3D predicts RNA 3D structures through the assembly of the 3D templates for secondary structural motifs (bulge, internal and multi-branched loops, shown in Supplementary Figure S1) and A-form helices. The criteria for template searching in Vfold3D is motif type, loop size and sequence dependent. If no templates can be found for the given secondary structural motifs, we break the motifs into single-stranded loops.
- To build all-atom structures for single-stranded loops using VfoldLA (39). VfoldLA builds RNA 3D structures through the assembly of 3D templates for single-stranded loops (hairpin, helix2 and tails, shown in Supplementary Figure S1) and A-form helices. The criteria for the template search in VfoldLA is loop type, size, and sequence dependent.
- To assemble structures from helices, motifs, and loops to build all-atom structures of the whole RNA, and refine the assembled structures using energy minimization, such as AMBER and NAMD, to remove the steric clash. Manual local structure adjustment may be applied to disentangle the strands (for loop strand penetrating helices) as a pre-simulation treatment. Here, we use the 5000-step NAMD energy minimization to refine the predicted all-atom structures, embedded in a water box, neutralized by Na^+^.

## RESULTS AND DISCUSSION

We use the 20 PK motifs and 7 KISS motifs listed in Supplementary Tables S1 and S2 to elucidate the topological constraints induced by the cross-linked base pairs, as compared with the constraints encoded in secondary structural motifs, and its potential applications to RNA 3D structure prediction.

### Pseudoknotted motifs

As shown in Supplementary Figure S4(A), the 20 pseudoknotted structures with different helix and loop sizes (H1–L1–L2–L3–H2, as listed in Table 1) show similar global folds, with the two helices (nearly) coaxially stacked. We use the Vfold coarse-grained conformational model to sample all the two-helix configurations using the discrete transformation matrix of (θ, φ, α, *dx*, *dy*, *dz*).

**Table 1.** Topological constraints of the PK motif, defined by the size of H1–L1–L2–L3–H2. We use loop L2 to exhaustively sample all the possible two-helix configurations in the MBB of H1–L2–H2. *F*_*x*_ is the fraction of the Vfold-allowed helical configurations with the corresponding loops. *F*_PK_ (*F*_123_) denotes the total topological constraints of a PK structure

PDB	Size	*F*_2_	*F*_12_	*F*_23_	*F*_PK_
1a60	3–4–0–3–5	5.2E–3	1.8E–4	2.0E–4	4.6E–5
1e95	6–1–0–9–6	3.2E–3	1.8E–6	2.9E–3	1.8E–6
1hvu	5–2–0–3–6	4.3E–3	6.5E–6	3.8E–5	1.2E–7
1ymo	6–8–0–8–9	3.2E–3	2.5E–3	2.2E–3	1.7E–3
2n6q	4–3–0–4–8	4.9E–3	3.9E–5	2.8E–4	9.2E–6
2tpk	5–1–0–7–7	4.3E–3	7.1E–8	1.5E–3	7.1E–8
4p5j	3–3–0–3–6	5.2E–3	4.2E–5	2.0E–4	2.1E–5
2m8k	5–6–0–5–12	4.3E–3	5.8E–4	2.4E–4	2.6E–5
2ap0	5–1–1–9–3	2.8E–2	1.0E–4	2.6E–2	1.0E–4
1kpd	5–3–1–7–5	2.7E–2	3.8E–4	9.6E–3	3.1E–4
1rnk	5–2–1–8–6	2.7E–2	6.3E–5	1.7E–2	6.3E–5
1yg4	5–2–1–9–3	2.8E–2	4.6E–4	2.6E–2	4.5E–4
2a43	4–2–1–9–3	3.1E–2	4.6E–4	3.0E–2	4.6E–4
2rp1	5–2–1–8–3	2.8E–2	4.6E–4	2.0E–2	3.8E–4
2xdd	4–4–1–6–3	3.1E–2	3.6E–3	1.0E–2	1.6E–3
4ato	3–3–1–5–4	3.2E–2	8.6E–4	6.9E–3	5.4E–4
437d	5–2–1–7–3	2.8E–2	4.6E–4	1.2E–2	2.3E–4
4rmo	5–3–2–9–5	5.6E–2	8.3E–4	4.2E–2	8.3E–4
1kaj	5–3–2–8–4	5.7E–2	1.6E–3	3.1E–2	1.4E–3
2m58	3–3–3–7–4	9.7E–2	3.3E–3	6.1E–2	3.0E–3
**Average**		**2.5E–2**	**7.9E–4**	**1.5E–2**	**5.6E–4**

**Figure 3.**
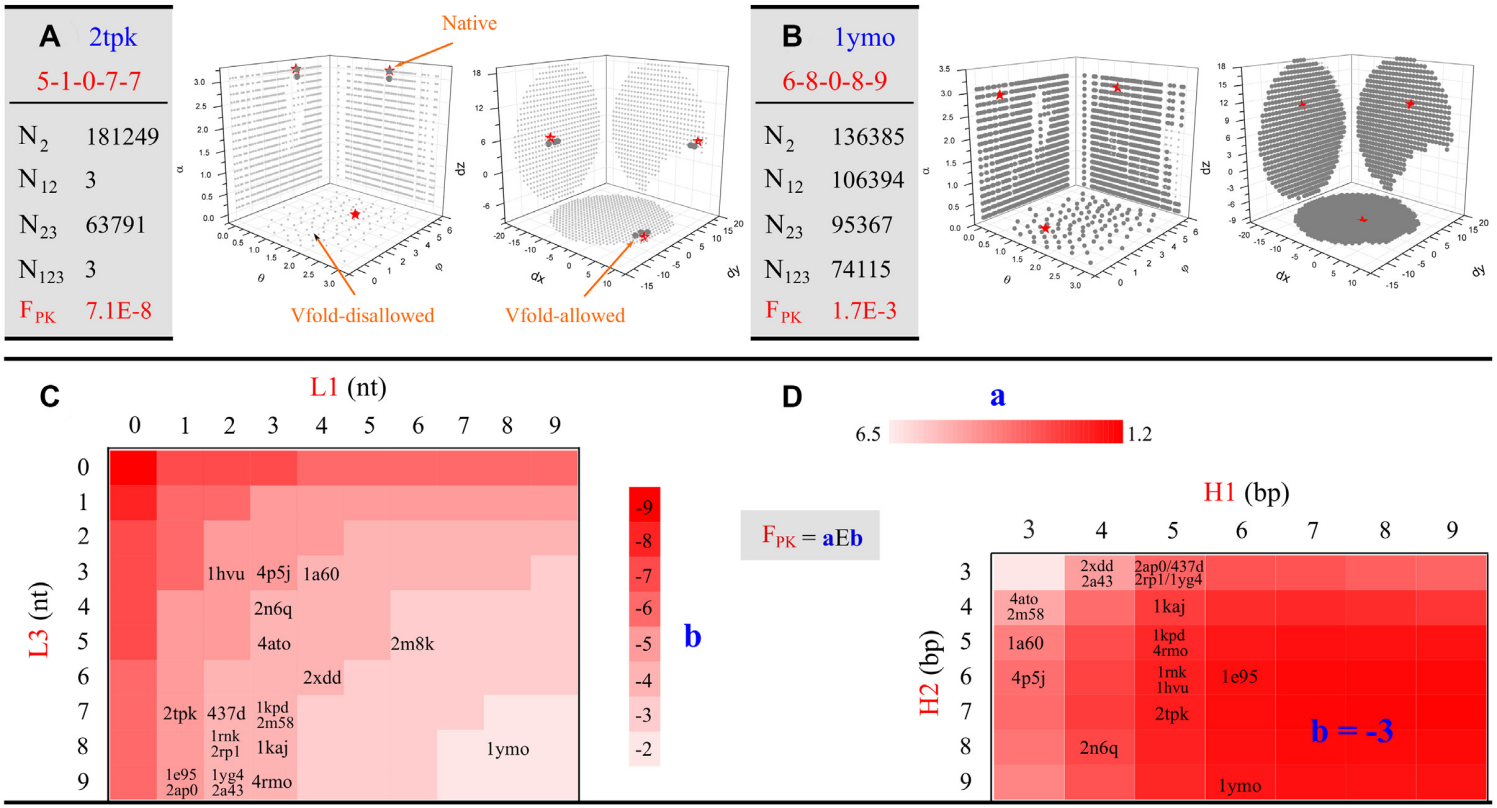
The quantification of the conformational sampling and the projections of the allowed (large dark gray) and disallowed (small light gray) helix configurations on six planes (three for translation, and three for rotation) for (**A**) 2tpk, and (**B**) 1ymo, respectively. The red stars are from the corresponding native structures. *N*_x_ is the number of the Vfold-allowed helix configurations with the respective loop connections. *F*_PK_ is the fraction of the Vfold-allowed helix configurations of a PK motif. (**C**) Loop L1 and L3 dependence of the topological constraints for PK, *F*_PK_(L1, L3), averaged over helices H1 and H2 (from 3 to 9 bp) and loop L2 (from 0 to 3 nt). The color bar is based on the value *b* of *F*_PK_. PDB IDs are placed according to (L1, L3). (**D**) Helix H1 and H2 dependence of the topological constraints for PK, *F*_PK_(H1, H2), averaged over loops L1 and L3 (from 0 to 9 nt) and loop L2 (from 0 to 3 nt). The color bar is based on the value *a* of *F*_PK_ (with *b* = –3). PDB IDs are placed according to (H1, H2).

As shown in Figure 3A and B and Supplementary Figures S6–S10, the pseudoknot topology imposes significant constraints on otherwise free helix orientations. For example, the 2tpk pseudoknot (5–1–0–7–7) in Figure 3(A) has (*N*_2_ =) 181 249 viable Vfold-allowed helical configurations of H1–L2–H2 in its MBB. The topological constraints of H1–L2–H2 (from loop L2) can be quantified, as listed in Table 1, by the fraction of *F*_2_ = *N*_2_/*N*$_{\rm tot}^{\rm MBB}$ = 4.3E–3. Here, the total possible helix configurations in MBB *N*$_{\rm tot}^{\rm MBB}$ = *R* × *T*, with *R* = 1800 for the rotations, and *T* = 23 548 (L2 = 0 nt) of the total grids in MBB for the translations. For each Vfold-allowed helical configurations of H1–L2–H2, we assess the topological constraints with additional loops by counting for the number of viable helical configurations. *N*_12_ ( = 3) and *N*_23_ (= 63791) gives the number of viable configurations for the H1–L2–H2 motif with loop L1 and L3, respectively. Since loop L1 (= 1 nt) is much shorter than L3 (= 7 nt), the topological constraint from L1 (from 4.3E–3 (of F_2_) to 7.1E–8 (of F_12_)) is much stronger than that from L3 (F_23_ =1.5E–3). Furthermore, *N*_123_ denotes the total number of viable helical configurations for the PK motif. The total topological constraints on 2tpk is F_PK_ = *N*_123_/*N*$_{\rm tot}^{\rm MBB}$ = 7.1E–8 with the major contributions from L1 and L2 loops.

For the 1ymo pseudoknot (6–8–0–8–9) shown in Figure 3(B), because of the relatively large size of loops L1 (= 8 nt) and L3 (= 8 nt), we find that the total topological constraints is *F*_PK_ = 1.7E–3, with the major contributions from loop L2 (= 0 nt) with F_2_ = 3.2E–3. On the other hand, 2m8k (5–6–0–5–12), similar to 1ymo, has large size of loops L1 and L3. However, 2m8k involves a much stronger constraint than 1ymo (*F*_PK_ = 2.6E–5 for 2m8k versus 1.7E–3 for 1ymo), suggesting the helix contribution to the overall topological constraints. From Table 1 of the 20 cases with loop L2 ranging from 0 to 3 nt in length, we find that the constraints from the L2 loop, F_2_, of H1–L2–H2 decreases with the length of L2 (the average of F_2_ for L2 = 0, 1, 2 and 3 nt are 4.3E–3, 2.9E–2, 5.6E–2 and 9.7E–2, respectively). Shorter L2 leads to stronger excluded volume effects between helices. Moreover, the asymmetrical A-form structure of helices may also contribute to the significant loop- and helix-size dependent of the helical configurations of a pseudoknot. The total topological constraints, *F*_PK_, ranges from E–3 to E–8 in magnitude, with 5.6E–4 in average, for the 20 cases.

To better reveal the fundamental principles of topological constraints encoded in PK, we compute the fraction *F*_PK_ for all PKs with H1 and H2 ranging from 3 to 9-bp, L1 and L3 from 0 to 9-nt, and L2 from 0 to 3-nt to calculate the averaged fraction of *F*_PK_(L1, L3), as shown in Figure 3(C). The magnitude of *F*_PK_ ranges from 1E-2 to 1E-9. Smaller L1 and L3 in size would cause stronger topological constraints (decreasing *b* in *F*_PK_). The overall shape of the ‘landscape’ might be funnel-like, with quite good symmetry with respect to L1 and L3. According to the sizes of 20 native PK structures, as listed in Table 1, we find that (i) most cases have the sizes of (L1, L3) with moderate averaged topological constraints (ranging from 1E-4 to 1E-6); (ii) larger size of L3 (compared with L1) is preferable. As listed in Table 1, the average *F*_12_ (= 7.9E–4) is much smaller than *F*_23_ (= 1.5E–2). Therefore, loops L1 and L2 play the major role in the topological constraints of the wild-type PKs.

Energetically, RNA topological constraint is related to the entropic contribution to RNA stability. Stronger topological constraints with shorter loops lead to a higher entropic folding free energy (and lower stability). At the 2D level, the enthalpic contribution of RNA stability is mainly from the helices (base pairs). Larger size of helices (depending on the sequences) can result in a lower enthalpic free energy (higher stability). Therefore, there is a balance between the sizes of loops and helices in RNA folding. As a compromise, the wild-type native structures in general involve moderate topological constraints. On the other hand, the tertiary interactions (depending on the sequences) at the 3D level, such as the coaxial stacking between H1 and H2, base triples between L3 and H2, and non-canonical interactions within loops, can further stabilize the structures while reducing the corresponding conformational space (strengthening the topological constraints). For instance, the strong topological constraints of 2tpk (*F*_PK_ = 7.1E–8 with very limited number of allowed helix configurations *N*_123_ = 3), as shown in Figure 3(A), may be counteracted by the stability from two helices and rich tertiary interactions.

Moreover, as shown in Figure 3D, we also calculate the averaged topological constraint fraction *F*_PK_(H1, H2). Compared with *F*_PK_(L1, L3) shown in Figure 3C, the ‘landscape’ becomes much flattened (all *F*_PK_ have the same magnitude of 1E–3). Larger H1 and H2 in size result in stronger topological constraints (decreasing *a* in *F*_PK_). However, for a given H1 (H2), there is a trend of decreasing then increasing in *a* with the elongation of H2 (H1). For example, 6.5 → 3.5 → 4.2 for the *a* value for H2 = 3, 7 and 9 bp, respectively, with H1 = 3 bp. This factor should be related to the helical structure of an A-form RNA helix. According to the sizes of native PKs, as listed in Table 1, we find that the wild-type PK structures prefer small sizes of H1 (<7-bp) with a weaker preference for H2.

Although the impact of the helix contribution on the topological fraction (averaged over different loops) is weaker than that of the loop contribution (Figure 3 C), the two helices and three loops in a PK motif are strongly coupled structurally by the loop-helix connectivity and loop-helix excluded volume effect in 3D. Such a coupling results in loop thermodynamic parameters dependent on both loop and helix sizes (41–44). In contrast, the loop parameter for a secondary structural motif, such as an internal loop, is mainly determined by loop itself. The structural coupling effects above become stronger for PKs with large helices and short loops, such as 2n6q and 2tpk.

### Hairpin-hairpin kissing motifs

As shown in Supplementary Figure S4(B-1), the hairpin–hairpin kissing motif involves at least three (H1, H2 and H3) helices and four (L1, L2, L4 and L5) loops, with the inter-strand hairpin–hairpin kissing base pairs of H2. Loop L3 would convert a two-strand KISS motif into a one-strand KISS motif with enhanced structural topological constraints. Furthermore, several RNA structures involve the kissing base pairs between the two hairpins within a multi-branched junction, as shown in Supplementary Figure S4(D-1) of the hairpin-hairpin kissing in a three-way junction. The loops La and Lb and the helix Hab may contribute weakly to the preference of the orientation of Hab, however, their constraints for the three-helix (H1–H2–H3) orientation may be much stronger. Here, we use the virtual loop L6, shown as the dotted orange line in Supplementary Figure S4(B-1), to denote the constraints from loops La and Lb and the helix Hab. In the following calculations, we substitute the helix Hab with two nucleotides, resulting in an effective loop length of L6: L6 = La + Lb + 2. The effective hairpin-hairpin kissing motif, described by the size of (H1–H2–H3)-(L1–L2–L3–L4–L5–L6), involves three helices and six loops.

As shown in Supplementary Figure S4(B-2), the superposition of the seven crystal KISS structures in Table 2 with different helix and loop sizes suggests that these structures share the similar global topology (helical configurations). Since the L2 and L4 loops are likely shorter than others, for a fixed H2 helix, we use the HLH motifs of H1–L2–H2 and H2–L4–H3 to efficiently sample the three-helix configurations in their corresponding MBB. For each three-helix configuration, as shown in Supplementary Figure S5, we use the Vfold model to sample the loop conformations and determine the topological constraints. We note that unlike a PK motif, a KISS motif needs two sets of (θ, φ, α, *dx*, *dy*, *dz*) to describe each configurations.

**Table 2.** Topological constraints of the KISS motif, defined by the size of (H1–H2–H3)-(L1–L2–L3–L4–L5–L6). We use loops L2 and L4 to randomly sample the three-helix configurations in the MBBs of H1–L2–H2 and H2–L4–H3. *F*_*x*_ is the fraction of the Vfold-allowed helical configurations with the corresponding loops. *F*_KISS_ (*F*_123456_) denotes the total topological constraints of a KISS structure

PDB	Size	*F*_24_	*F*_124_	*F*_234_	*F*_245_	*F*_246_	*F*_KISS_
1e8o	(7–2–4)–(3–1–4–1–5–2)	8.5E–4	6.0E–5	3.9E–5	3.6E–5	2.7E–5	1.4E–7
3skl	(6–2–7)–(5–0–8–0–2–7)	2.3E–5	9.4E–6	6.7E–6	4.8E–7	1.2E–5	4.4E–8
3ds7	(7–2–6)–(5–0–8–0–5–7)	2.0E–5	8.8E–6	6.8E–6	9.4E–6	1.5E–5	1.0E–6
3ivn	(6–2–5)–(6–1–8–1–6–7)	9.4E–4	6.5E–4	2.8E–4	7.2E–4	7.1E–4	1.3E–4
4lx5	(6–2–6)–(6–1–8–0–5–7)	1.5E–4	9.9E–5	4.2E–5	7.5E–5	9.8E–5	1.1E–5
4wfl	(4–5–5)–(4–2–3–3–3–2)	5.2E–3	1.7E–4	4.0E–5	9.4E–5	3.7E–5	9.9E–11
4uyk	(5–5–4)–(3–1–4–4–5–2)	3.4E–3	4.1E–5	8.2E–5	3.3E–4	4.1E–5	2.5E–9

As listed in Table 2, the contributions from the L2 and L4 loops, *F*_24_, ranges from E–3 to E–5 in magnitude. Shorter loop size, as expected, creates stronger constraints. For example, 4wfl and 4uyk (with longer L2 and L4) involve weaker constraints. The contributions from other four loops, i.e. *F*_124_, *F*_234_, *F*_245_ and *F*_246_, also show the loop size and helix size dependence of the topological constraints. *F*_245_ (= 4.8E–7) of 3skl, with L5 = 2 nt, shows a stronger constraint than that from other three loops. Because of the longer loop sizes of 3ds7, 3ivn, and 4lx5, the contribution from all four loops (*F*_24*x*_/*F*_24_) are E–1 (much weaker constraint). On the other hand, a longer kissing helix, such as the 5-bp kissing helix in 4wfl and 4uyk, results in a stronger topological restriction (*F*_24*x*_/*F*_24_ ranges from E–2 to E–3 for all four loops). This is because the formation of the kissing helix H2 in a KISS motif tends to rigidify the two originally flexible hairpin loops. The total topological constraints, *F*_KISS_, ranges from 1E–4 to 1E–11 for the seven cases.

A KISS motif contains four looping circuits: L1–H2 (one chain)-L2–H1 (terminal base pair), L4–H2 (one chain)-L5–H3 (terminal base pair), L2–H1 (one chain)-L3–H3 (one chain)-L4–H2 (terminal base pair), and L3–H3 (terminal base pair)-L6–H1 (terminal base pair), as shown the red dotted lines in Supplementary Figure S1. It is the simultaneous effects of the four ‘loops’, in addition to the helix formation and the resultant excluded volume and chain rigidity effects, that contribute to the total topological constraints of a KISS motif. For a PK motif in Supplementary Figure S1, there are only two circuits: L1–H2 (one chain)-L2–H1 (terminal base pair) and L2–H1 (one chain)-L3–H2 (terminal base pair), thus, the topological constraints on average are weaker than the KISS motif. On the other hand, a secondary structural motif (hairpin, internal, and bulge loops and multi-branched junctions) involves only one circuit, and shows much weaker topological constraints than a tertiary structural motif.

### Extended kissing motifs

The generalized hairpin-hairpin kissing motif can be extended to the following more complicated kissing motifs, involving multiple helices and loops. Supplementary Figure S4(C-1) gives a hairpin-internal loop kissing motif. Supplementary Figure S4(D-1) denotes a hairpin loop kissed by a stem-loop terminal loop. Due to the extremely long computational time, we do not perform the exhaustive conformational sampling and comprehensive analysis for the topology constraints for these two extended kissing motifs. It is important to note that as shown in Supplementary Figure S4(C-2 and D-2), the crystal structures of the corresponding RNAs are similar to each other, despite the different helix and loop sizes and different sequence (listed in Supplementary Tables S3 and S4).

Here we use the RNApdbee (45) to extract the 2D structures for all the cases of the two extended kissing motifs listed in Supplementary Tables S3 and S4. Although some base pairs are not treated as standard canonical base pairs in RNApdbee, according to the global structures shown in Supplementary Figures S4(C-2 and D-2), we manually categorize them into the above two kissing motifs. The small perturbation in the sequence leads to slight changes in 2D structures, such as the different sizes of loops and helices. Such changes would subsequently cause changes in the conformational space. However, the structure similarity of the above two extended KISS motifs, as well as the PK and KISS motifs indicates that RNAs with same tertiary structural motifs may conserve their global structural folds with the tolerance of slight changes in the sizes of loops and helices, which raises a potential application for RNA 3D structure prediction.

### Application to RNA structure prediction

Despite the rapid development of various computational models for RNA structures (17–39), accurate and efficient predictions for RNA tertiary folds remain a significant challenge and impact our mechanistic understanding of RNA functions. Knowledge-based RNA 3D structure prediction methods, such as MC-sym, Vfold3D, RNAcomposer, are severely limited by the completeness of the motif templates database. Due to the limited numbers of known RNA 3D structures in PDB database, currently, the low success rate of finding a proper template for a given motif, especially for the motifs with cross-linked contacts, hampers predictions of large RNAs with tertiary base pairs. From our analysis of the structural topological constraints for the PK and KISS motifs, which concludes that the tertiary base pairs can significantly reduce the conformational space, we develop a topology-directed RNA 3D structure prediction approach to overcome the limitation of the motif template-based prediction methods.

**Figure 4.**
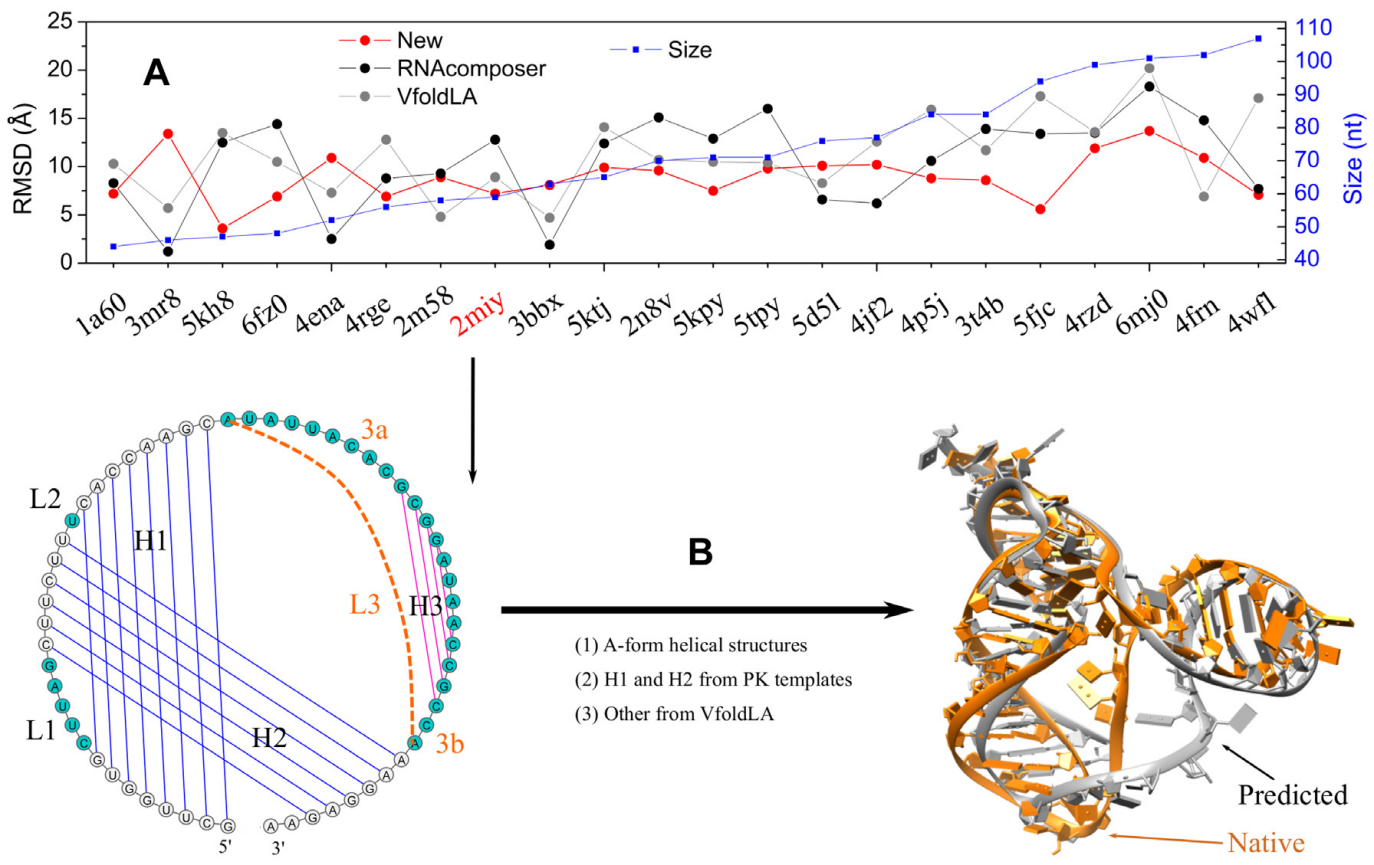
(**A**) Comparison of the RMSDs between RNAcomposer, VfoldLA, and the current new model. The sequences and 2D structures of all the test RNAs are listed in supplementary Table S5. (**B**) Topology-based RNA 3D structure prediction for the case of 2miy, a PK with substructure. The method integrates tertiary structural motif templates for helix orientation, Vfold3D for secondary structural motifs, and VfoldLA for single-stranded loop structures.

As a benchmark test, we compare the new multi-stage model with RNAcomposer and VfoldLA servers for 22 RNAs as shown in Figure 4(A). The comparisons are based on the same 2D structures provided as input. The corresponding 2D structures are extracted from the native 3D structures (see Supplementary Table S5 for details). All the tested RNAs contain cross-linked base pairs of different structural complexity, with the sizes ranging from 44 to 107 nt. To avoid using templates from the native structures, we exclude all the native structures from the template database. For RNAcomposer, we upload the native structure to exclude native 3D structure elements and set the maximum number of 3D models for each prediction to be ten. For VfoldLA, we use the five clusters given by the default output. The model with the minimum heavy-atom RMSD is selected as the predicted structures for each test case.

As shown in Figure 4A, our model gives the low-resolution predictions for most tested RNAs, with 14 (out of 22) cases having RMSDs in the range of (5.0, 10.0) Å. One RNA (PDB: 5kh8) achieves the near-atomic prediction with the RMSD < 5.0 Å, while seven of them have large RMSDs (>10.0 Å). The average RMSD is 8.9 Å for the 22 tested cases. RNAcomposer and VfoldLA have the similar performance, with the average RMSDs of 10.6 Å and 11.2 Å, respectively. Although the native 3D structure elements are excluded from predictions, RNAcomposer can still reach the atomic resolution (< 3.0 Å) for three cases. However, there are 13 (15) tested cases with large RMSDs (>10.0 Å) from RNAcomposer (VfoldLA) predictions. As shown in Figure 2, our model builds the helix configurations of tertiary motifs (involving cross-linked base pairs) based on the templates of known structures. The better treatment of the topological constraints induced by the cross-linked base pairs, such as PK and KISS motifs, may contribute to the improved accuracy.

As shown in Figure 4B, the preQ1 Class II riboswitch from *Streptococcus pneumoniae* (PDB: 2miy) (46), is a pseudoknotted structure with a hairpin substructure in loop L3. By substituting the hairpin substructure with two nucleotides in loop L3, we convert its 2D structure into a PK motif attached by a 2-nt 3′ tail and a hairpin structure (helix H3 and a 4-nt hairpin loop) with flanking 9-nt and 2-nt tails on two sides. We build the two-helix (H1 and H2) orientation based on a PK motif template (1yg4), which has the similar loop and helix sizes. We then predict the structures for the hairpin and all the loops using VfoldLA. As shown the 3D structures in Figure 4B, the predicted structure preserves the coaxial stacking interactions between H1 and H2. Compared with the VfoldLA predictions, the improved accuracy (7.2 Å from the current new model versus 8.9 Å from VfoldLA) stems from the better prediction of the helix configuration (H1 and H2) for the PK motif. In fact, the structure of the hairpin substructure (with weak topological constraints) is determined mainly by the interactions within loops (3a and 3b shown in Figure 4B) and between loops and helices. The prediction based on single-stranded loop sequences (VfoldLA) may not completely preserve the required interactions, leading to the (slightly) misplaced helix H3. Therefore, additional information, such as the tertiary interactions (contacts), may be needed to further improve the prediction.

**Figure 5.**
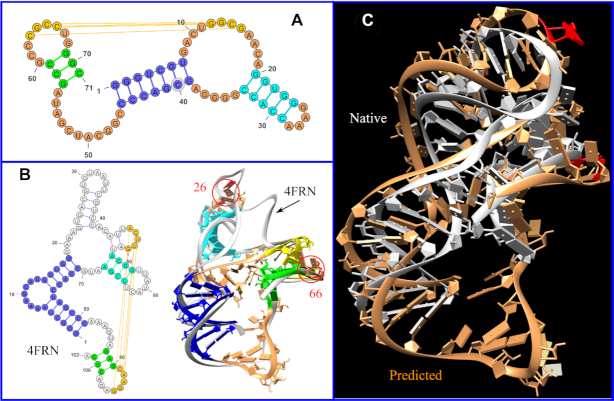
Topology-based RNA 3D structure prediction. (**A**) The 2D structure used for challenge #13 of *RNA Puzzles*, which contains four helices and seven loops. The helix in yellow is a hairpin-internal loop kissing helix. (**B**) The 2D and 3D (white ribbon) structures of the cobalamin riboswitch regulatory element (PDB: 4frn). We use the same color to show the alignment of the four helices in (A) to the helix parts of 4frn in (B). Since the two helices linked by a bulge loop, shown as the blue part of 4frn, are actually coaxially stacked in 3D, we consider the two helices as one effective helix. We model the four helices in (A) as A-form helices and place them according to the 3D topology of 4frn as shown in the right panel of (B). The 3D structures of the seven loops in (A) are assembled by VfoldLA. (**C**) The comparison between the predicted (in brown) and native (in white) structures (all-heavy-atom RMSD is 5.9 Å). We refine the position of helix in cyan according to the tertiary contact between A26 and C66 derived from the MOHCA data.

In addition, we use Challenge #13 of *RNA puzzles* as another example to illustrate our multi-stage method for RNA 3D structure prediction. *RNA Puzzles* is a CASP-like blind test and critical assessment of RNA structure prediction algorithms (17–19). Challenge #13 is for the structure of a 71-nt RNA sequence. To predict the 3D structure, we first predict the 2D structure using Vfold2D (38) with the information from Rfam (47). As shown in Figure 5A, the predicted 2D structure contains four helices and seven loops with a hairpin-internal loop kissing structure. From the known RNA 3D structures in the PDB database, we found that the crystal structure of the cobalamin riboswitch regulatory element (48) (PDB: 4frn) has a similar topology as the predicted 2D structure, with the two blue helices coaxial stacked and two additional helices (the two-bp helix can be considered as the intra-junction interactions). Therefore, as shown in Figure 5B, we placed four A-form helices according to the positions of the corresponding helices in the crystal structure 4frn. Furthermore, based on the MOHCA data (available at https://rmdb.stanford.edu), we refined the position of the helix in cyan to accommodate the tertiary contact between the nucleotides A26 and C66 (shown in red color in Figure 5B). Finally, we predicted all the loop structures using VfoldLA. The predicted structure shown in Figure 5C has the RMSD of 5.9 Å.

As indicated in the preceding section, the three helices of the hairpin-hairpin kissing motif are topologically coupled due to the loop–helix linkage. Therefore, based on the crystal structure of 4frn with the similar topology, we can predict the helix orientation (of the blue, green, and yellow helices shown in the figure). However, the additional helix (in cyan), which is one of the helices in the internal loop, has weak topological coupling with other helices and loops. The usage of the information of the tertiary interactions from the MOHCA data can improve the prediction accuracy.

## CONCLUSIONS

Through a systematic analysis for the topological constraints in pseudoknot (PK) and kissing (KISS) motifs, we propose a topology-based RNA 3D structure prediction method. Using the Vfold model to construct three-dimensional maps for the helix orientations, we find that the Vfold-accessible conformational space for tertiary structural motifs, such as PK and KISS, is dominantly determined by the motif type, helix size and loop size, indicating a strong structural (topological) coupling between helices and loops in RNA tertiary motifs. The similar global folds of the native structures, as shown in Supplementary Figure S4, suggest that the native structure may be predominantly determined by the topological constraints encoded at the 2D structure level.

Based on the dominant role of tertiary contacts (cross-linked base pairs) in determining the global topology of RNA 3D structure, we develop a topology-based RNA 3D structure prediction algorithm. The approach incorporates VfoldLA for single-stranded loops, Vfold3D for secondary structural motifs and tertiary structural motifs. The key ingredient of the structure prediction method is to build global scaffold (helix orientations) based on the homologous topology, predict component structures for loops and secondary structural motifs using previously developed models, and refine the structures with additional tertiary contacts information if available. This new method gives improved predictions for RNAs with tertiary (cross-linked) base pairs. However, similar to other template-based RNA structure prediction models, the method developed here cannot predict structures of ‘new’ topology. With the increasing number of the known RNA structures, the larger and more divergent pools of known structural topologies would lead to better predictions from the topology-based method. Further improvement of the model includes: (I) building an RNA topology database, which can accommodate non-canonical base pairs and different internal/bulge loops within helices; (II) improving the size similarity-based scoring scheme to better search for the optimal topologies in the database; (III) developing an all-atom scoring function to better select predicted structures when multiple templates are available.

## Supplementary Material

gkaa463_Supplemental_File
