# Supplementary material for: Topological constraints of RNA pseudoknotted and loop-kissing motifs: applications to three-dimensional structure prediction
Source: Nucleic Acids Res. 2020 Jun 3;48(12):6503–12. doi: 10.1093/nar/gkaa463 (PMC7337929; doi:10.1093/nar/gkaa463)
Supplement: gkaa463_Supplemental_File [file gkaa463_supplemental_file.pdf]

Supplementary Information:

Topological constraints of RNA pseudoknotted and loop-kissing motifs: applications to three-dimensional structure prediction

Xiaojun XU[1,‡] Shi-Jie CHEN[2,‡]

[1]Institute of Bioinformatics and Medical Engineering, Jiangsu University of Technology, Changzhou, Jiangsu 213001, China

[2]Department of Physics, Department of Biochemistry, and Informatics Institute, University of Missouri, Columbia, MO 65211, USA

[‡]Author to whom correspondence should be addressed; E-mail: chenshi@missouri.edu; xuxiaojun@jsut.edu.cn.

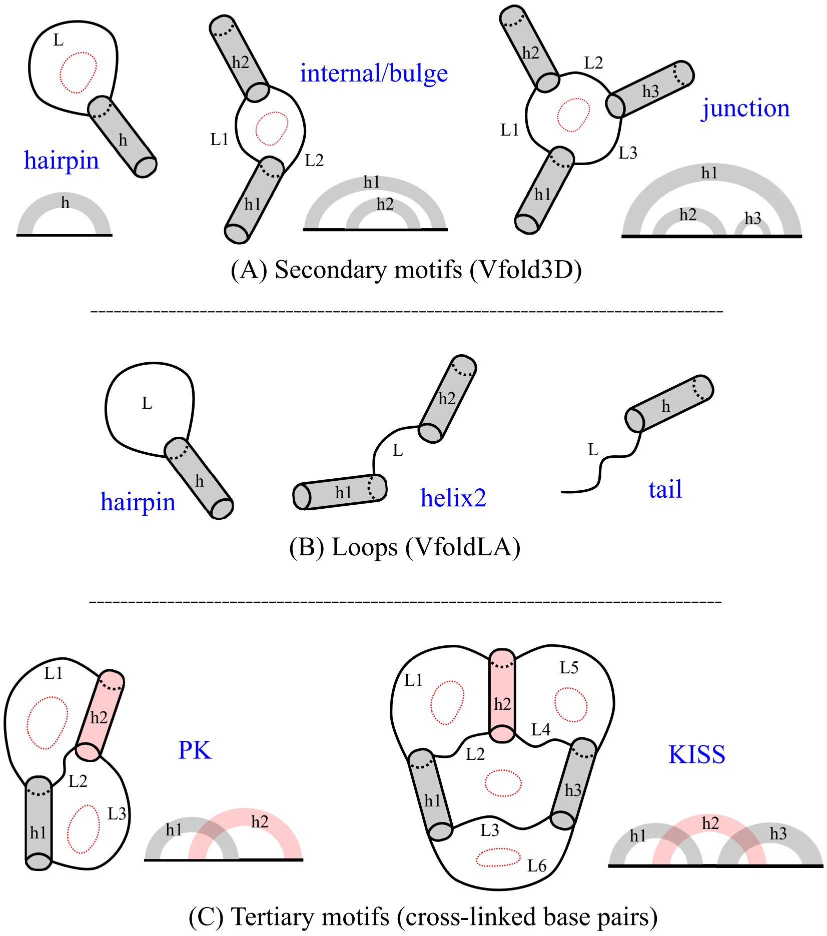

Figure S1: Definition of the RNA motifs: (A) secondary structural motifs of hairpin, bulge, internal loop, and multi-branched junction; (B) single-stranded loops of helix2, tail and hairpin. (C) tertiary structural motifs of PK and KISS, involving cross-linked base pairs; The red dotted lines denote the looping circuits within motifs. For example, the KISS motif has four looping circuits, while the secondary structural motifs only have one looping circuit.

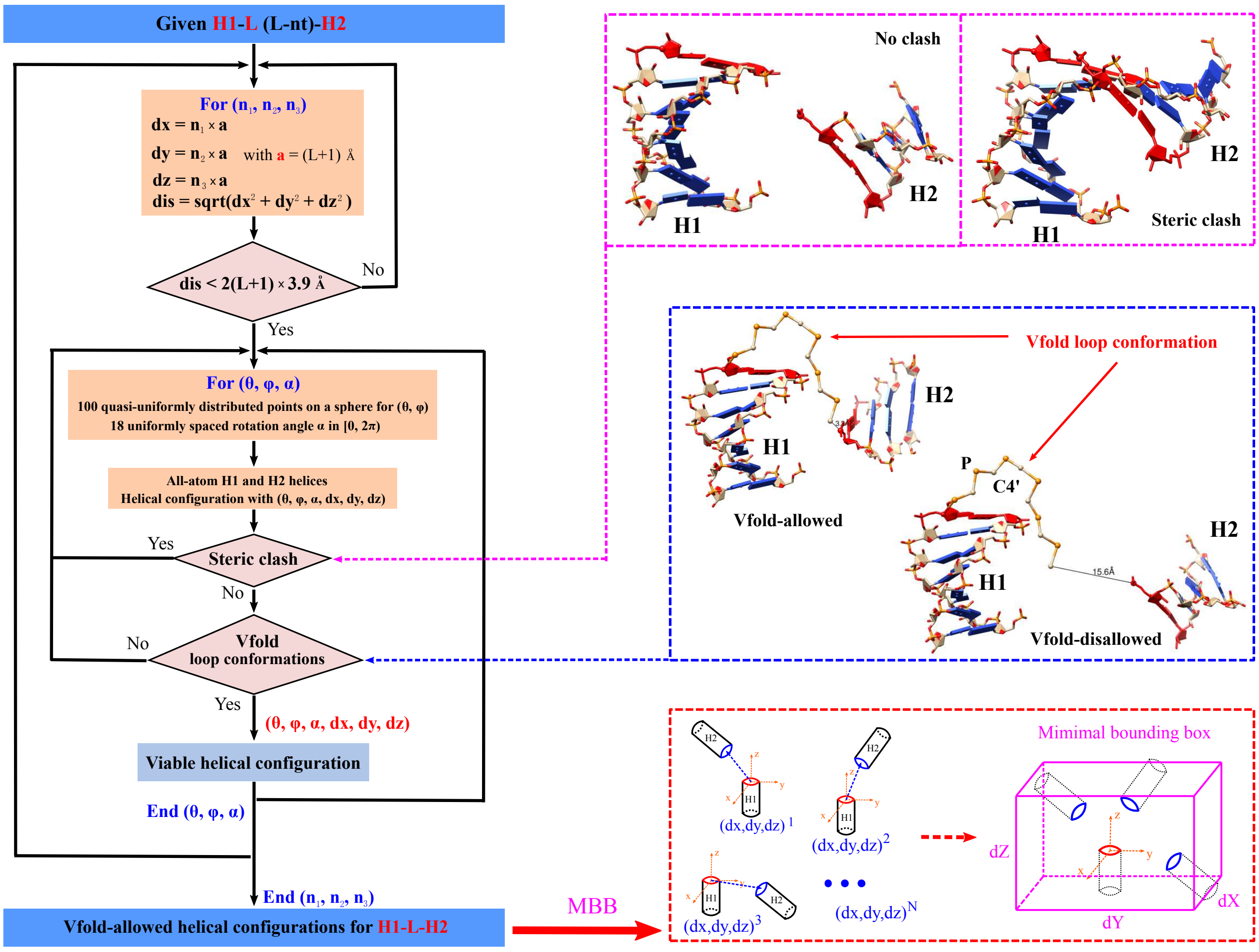

Figure S2: Calculation of the minimal bounding box (MBB) for HLH. a = (L +1) Å is the loop-size dependent adaptive grid size. The conditional statement of "dis $<$ 2(L+1) $\times$ 3.9 Å" ensures that the sampling of (dx, dy, dz) for the displacement is within a (loop-size dependent) reasonable region. We use the all atom A-form helical structures for helices H1 and H2 to account for the steric clash between helices. We calculate all the atom pair-wise distances between helices and consider the steric clash if the number of atom pairs with their distances less than 2.0 Å $>$ a given threshold (here, we use 5). For each helical configuration without steric clash between helices, we use Vfold to generate the virtual-bond (P-C4'-P) loop conformations (starting from H1) and calculate the distances between the atom C4' of the loop end and the atom P of the corresponding nucleotide in H2. If the distance is within the region of (3.9 - 1.9, 3.9 + 1.9) Å, the sampled loop is compatible with the given helical configuration, and the corresponding helical configuration is considered as a Vfold-allowed helical configuration for the HLH motif. Otherwise, it is Vfold-disallowed, if none of the sampled loop conformations meets the criteria. The geometric center of the terminal base pair in H2 (dx, dy, dz) of all the vfold-allowed configurations sweeps out a 3D region. The minimum box that contains such a region defines the MBB. Vfold model uses two bonds (P-C4' and C4'-P) to represent each nucleotide in loops, and enumerates loop backbone conformations with the bond length of 3.9 Å, and bond angle of $\sim$109.5°. Differed from the previous treatment for the loop backbone flexibility, we use the loop-size dependent torsional angles to sample the loop conformations. Specifically, we use ten, six, five, four, and three uniformly spaced rotation angles α between 0 and 2π to sample the loop conformations for the loops of size 1-nt, 2-nt, 3-nt, 4-nt, and $\geq$ 5-nt, respectively.

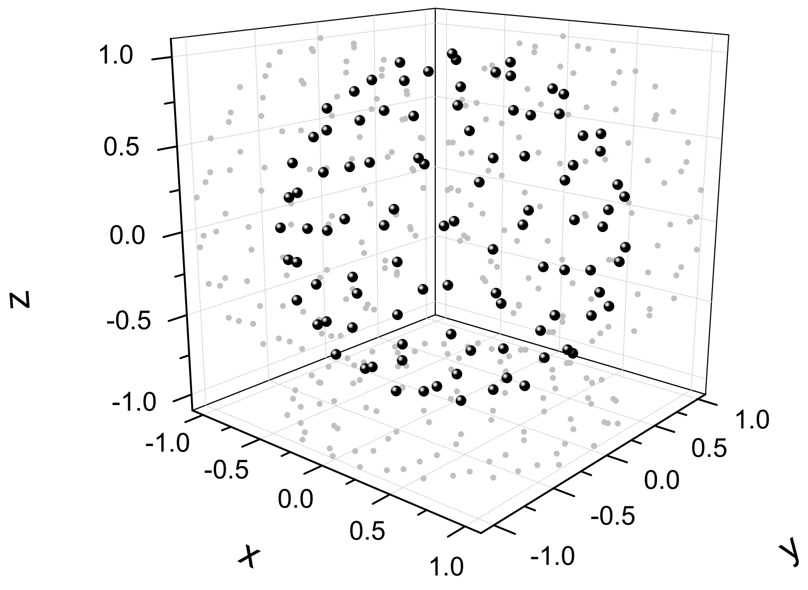

Figure S3: 100 quasi-uniformly distributed points on the surface of a sphere, generated by the Monte Carlo simulations. During the MC simulations, we randomly move particles on the surface of a sphere to minimize the total Lennard-Jones potential.

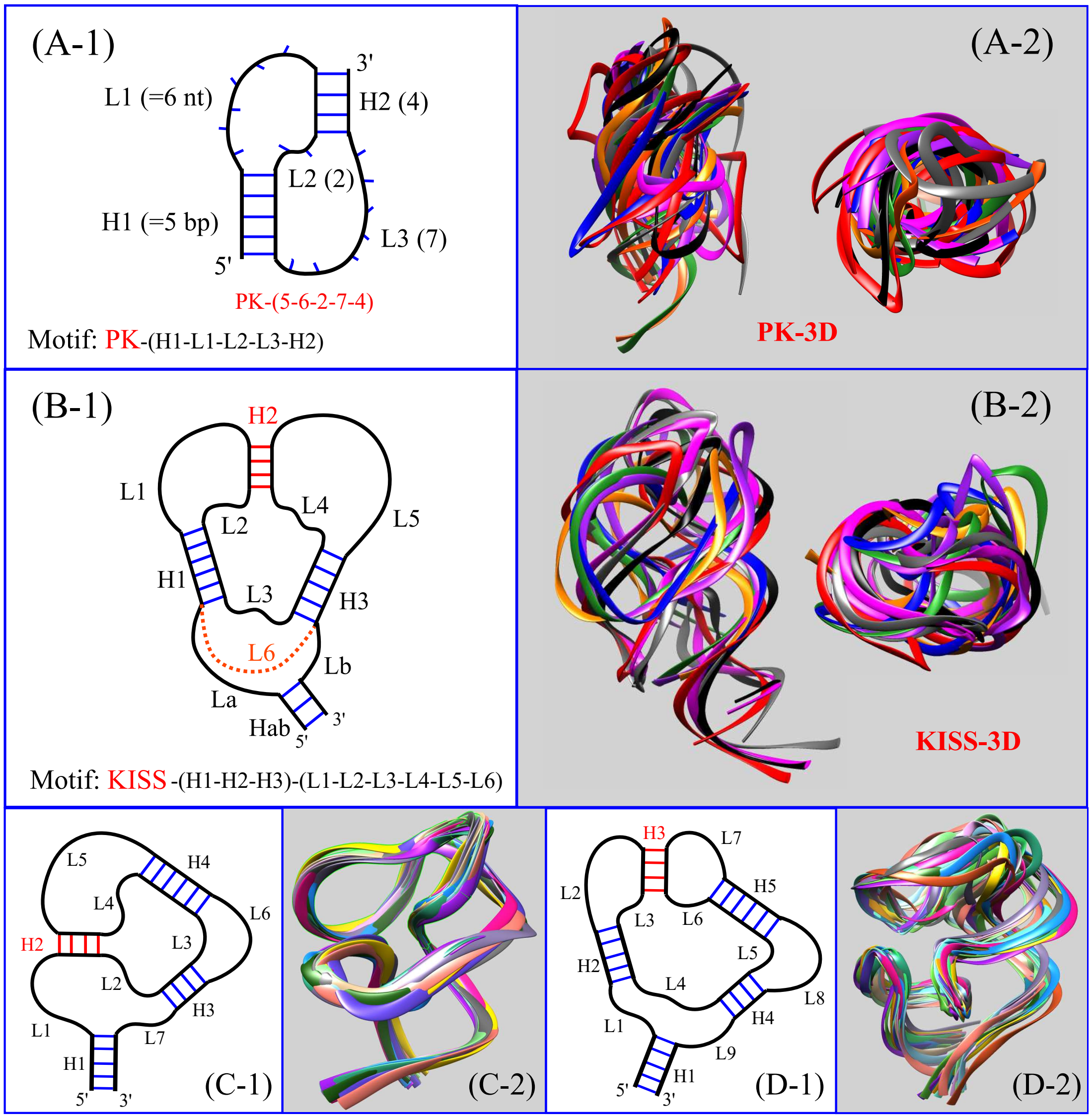

Figure S4: (A) The 2D and 3D (side- and top-view) structures of the PK motif, defined by the size of (H1-L1-L2-L3-H2), which contains two helices and three loops. Helices and loops are denoted by the numbers of base pairs and unpaired nucleotides, respectively. (B) The 2D and 3D (side- and top-view) structures of the KISS motif, defined by the size of (H1-H2-H3)-(L1-L2-L3-L4-L5-L6), which contains three helices and six loops. We introduce a virtual loop, L6, to effectively consider the influence of the L3-H3(bp)-L6-H1(bp) circuit in the topology constraints of the generalized hairpin-hairpin kissing motif, while omitting the additional freedom from the Hab helix. The size of L6 = La + Lb + 2. The kissing helix H2 (in red) locks the structure to the same global topology despite the sizes of the helices and the loops, as shown the 3D KISS structures in (B-2). The 2D and 3D structures of two extended kissing motifs: (C) hairpin-internal loop kissing; (D) hairpin-stem loop terminal loop kissing. Small changes in sequence lead to slight changes in the 2D structures, such as the different sizes of loops and helices. However, the kissing helices H2 in (C-1) and H3 in (D-1) (in red) lock the structures to the same topology, as shown in (C-2) and (D-2), indicating that it is the topology constraints and the sequence-dependent interactions that select specific/functional conformations within the allowed conformational ensemble. All 3D structures are in ribbon representation for clarity.

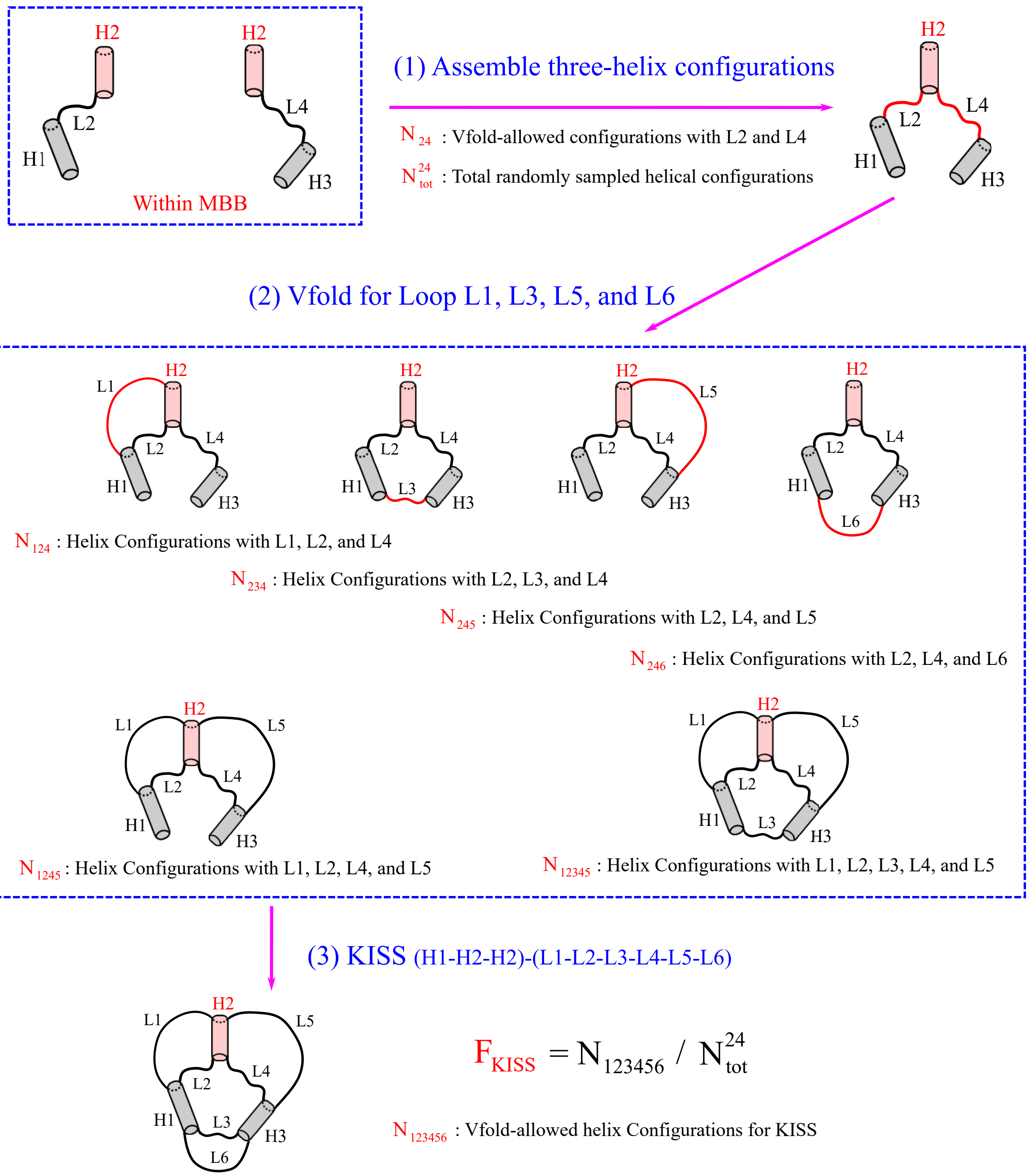

Figure S5: Topological constraints for KISS, which contains three helices and six loops. (1) We randomly assemble the three-helix configurations with the HLHs of H1-L2-H2 and H2-L4-H3 in the corresponding MBBs. N_{24} and N^{24}_{tot} are the numbers of the Vfold-allowed and total randomly sampled helix configurations, respectively. (2) For each of the Vfold-allowed (H1-H2-H3)-(L2-L4) configuration, we use Vfold to generate the virtual-bond structures of loop L1, L3, L5, and L6, resulting in the topological constraints of different loop connections. N_x is the number of Vfold-allowed three-helix configurations with the respective loop connections. (3) The fraction of $F_{KISS} = N_{123456} / N^{24}_{tot}$ defines the total topological constraint of the KISS motif.

(A-1) 1a60	
3-4-0-3-5	
R	1800
T	23548
N_2	219584
N_{12}	7675
N_{23}	8344
N_{123}	1956
F_{PK}	4.6E-5

(A-2)

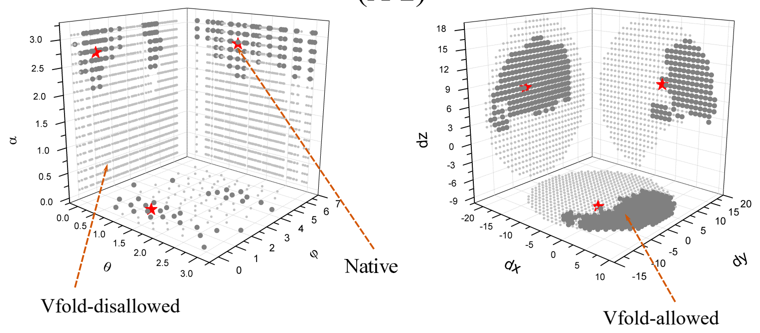

(B-1) 1e95	
6-1-0-9-6	
R	1800
T	23548
N_2	136385
N_{12}	78
N_{23}	124783
N_{123}	78
F_{PK}	1.8E-6

(B-2)

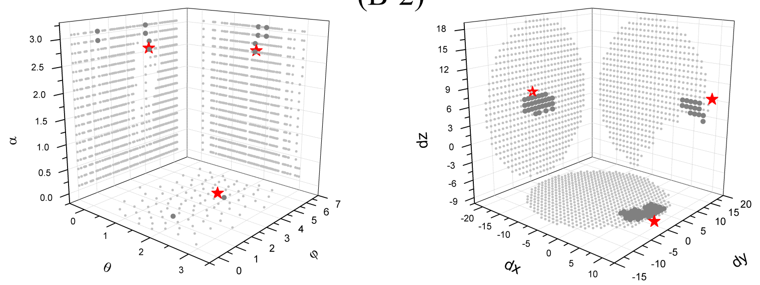

(C-1) 1hvu	
5-2-0-3-6	
R	1800
T	23548
N_2	181249
N_{12}	275
N_{23}	1611
N_{123}	5
F_{PK}	1.2E-7

(C-2)

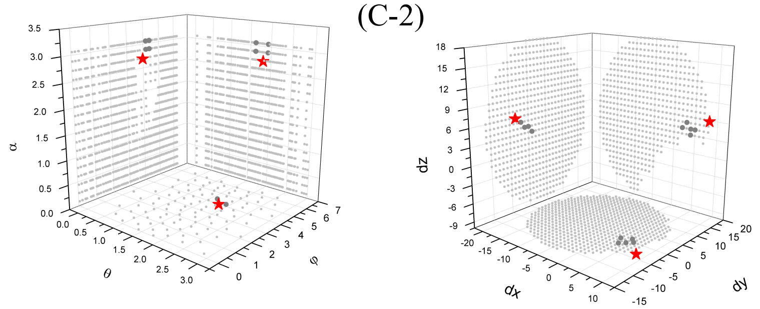

(D-1) 1ymo	
6-8-0-8-9	
R	1800
T	23548
N_2	136385
N_{12}	106394
N_{23}	95367
N_{123}	74115
F_{PK}	1.7E-3

(D-2)

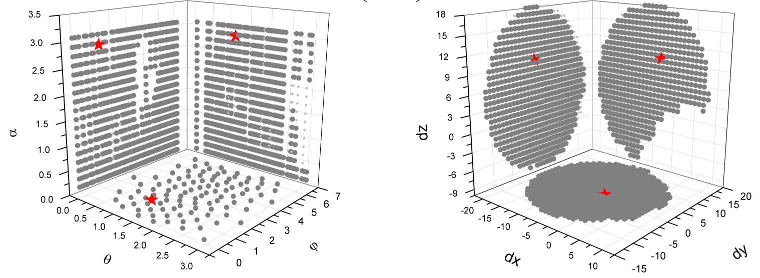

Figure S6: The quantification of the conformational sampling and the projections of the allowed (large dark gray) and disallowed (small light gray) helix configurations on six planes (three for translation, and three for rotation) for (A) 1a60, (B) 1e95, (C) 1hvu, and (D) 1ymo, respectively.

(A-1) 2ap0	
5-1-1-9-3	
R	1800
T	8820
N_2	449628
N_{12}	1664
N_{23}	414074
N_{123}	1664
F_{PK}	1.0E-4

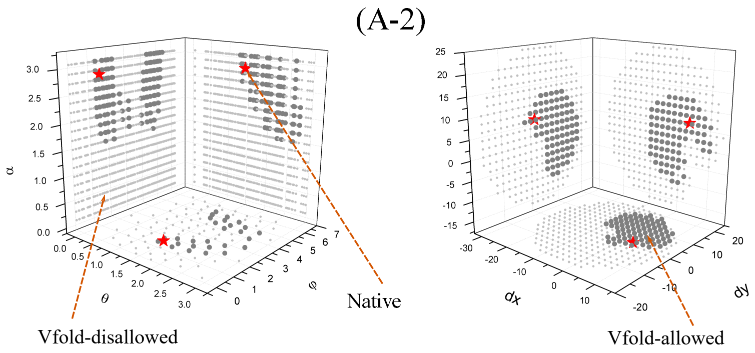

(B-1) 2n6q	
4-3-0-4-8	
R	1800
T	23548
N_2	206614
N_{12}	1665
N_{23}	11977
N_{123}	391
F_{PK}	9.2E-6

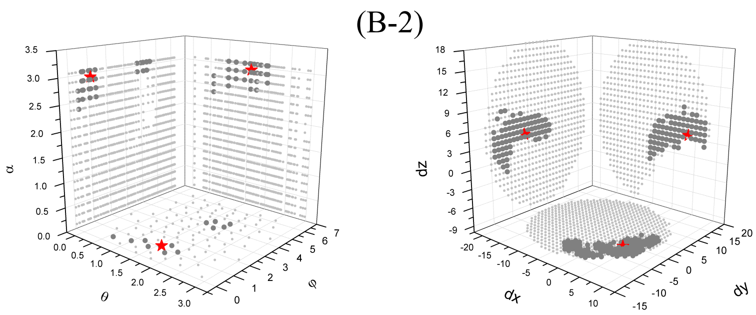

(C-1) 2tpk	
5-1-0-7-7	
R	1800
T	23548
N_2	181249
N_{12}	3
N_{23}	63791
N_{123}	3
F_{PK}	7.1E-8

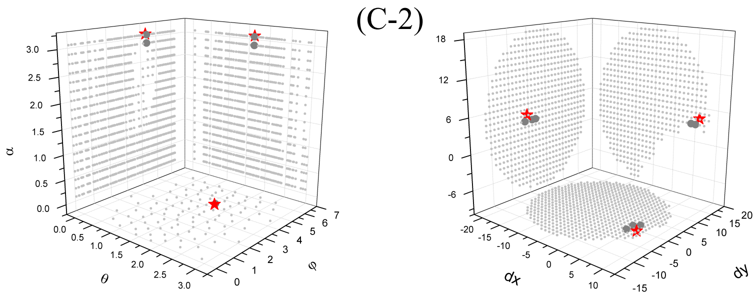

(D-1) 4p5j	
3-3-0-3-6	
R	1800
T	23548
N_2	219576
N_{12}	1799
N_{23}	8344
N_{123}	876
F_{PK}	2.1E-5

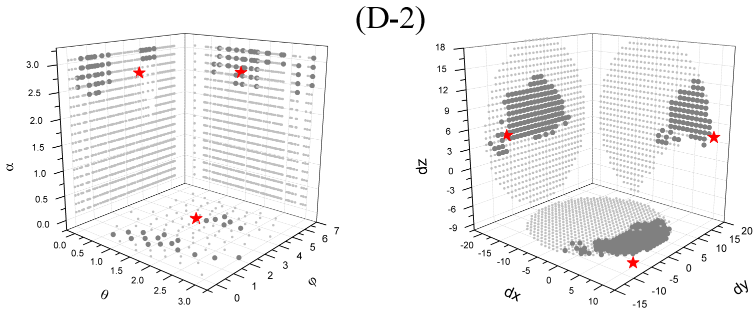

Figure S7: The quantification of the conformational sampling and the projections of the allowed (large dark gray) and disallowed (small light gray) helix configurations on six planes (three for translation, and three for rotation) for (A) 2ap0, (B) 2n6q, (C) 2tpk, and (D) 4p5j, respectively.

(A-1) 1kpd	
5-3-1-7-5	
R	1800
T	8820
N_2	431544
N_{12}	6020
N_{23}	153079
N_{123}	4931
F_{PK}	3.1E-4

(A-2)

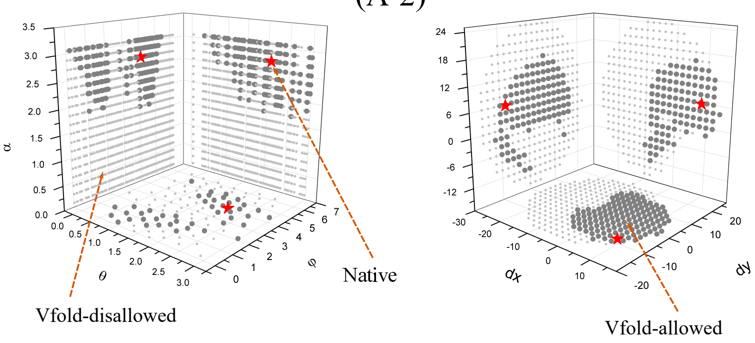

(B-1) 1rnk	
5-2-1-8-6	
R	1800
T	8820
N_2	431003
N_{12}	999
N_{23}	273253
N_{123}	999
F_{PK}	6.3E-5

(B-2)

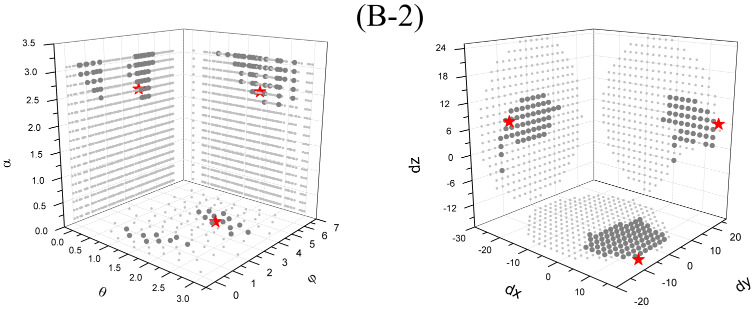

(C-1) 1yg4	
5-2-1-9-3	
R	1800
T	8820
N_2	449628
N_{12}	7265
N_{23}	414074
N_{123}	7200
F_{PK}	4.5E-4

(C-2)

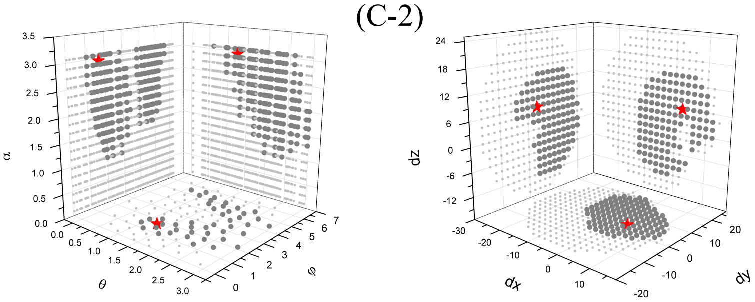

(D-1) 2a43	
4-2-1-9-3	
R	1800
T	8820
N_2	495024
N_{12}	7347
N_{23}	486598
N_{123}	7347
F_{PK}	4.6E-4

(D-2)

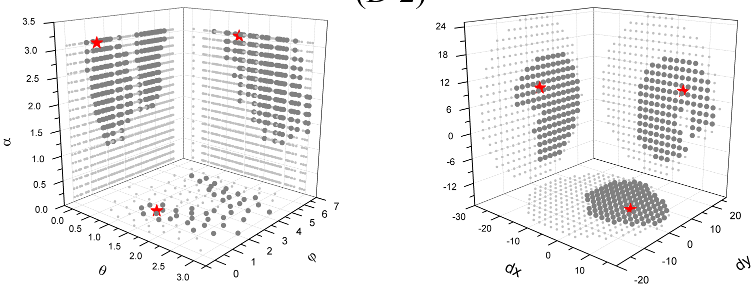

Figure S8: The quantification of the conformational sampling and the projections of the allowed (large dark gray) and disallowed (small light gray) helix configurations on six planes (three for translation, and three for rotation) for (A) 1kpd, (B) 1rnk, (C) 1yg4, and (D) 2a43, respectively.

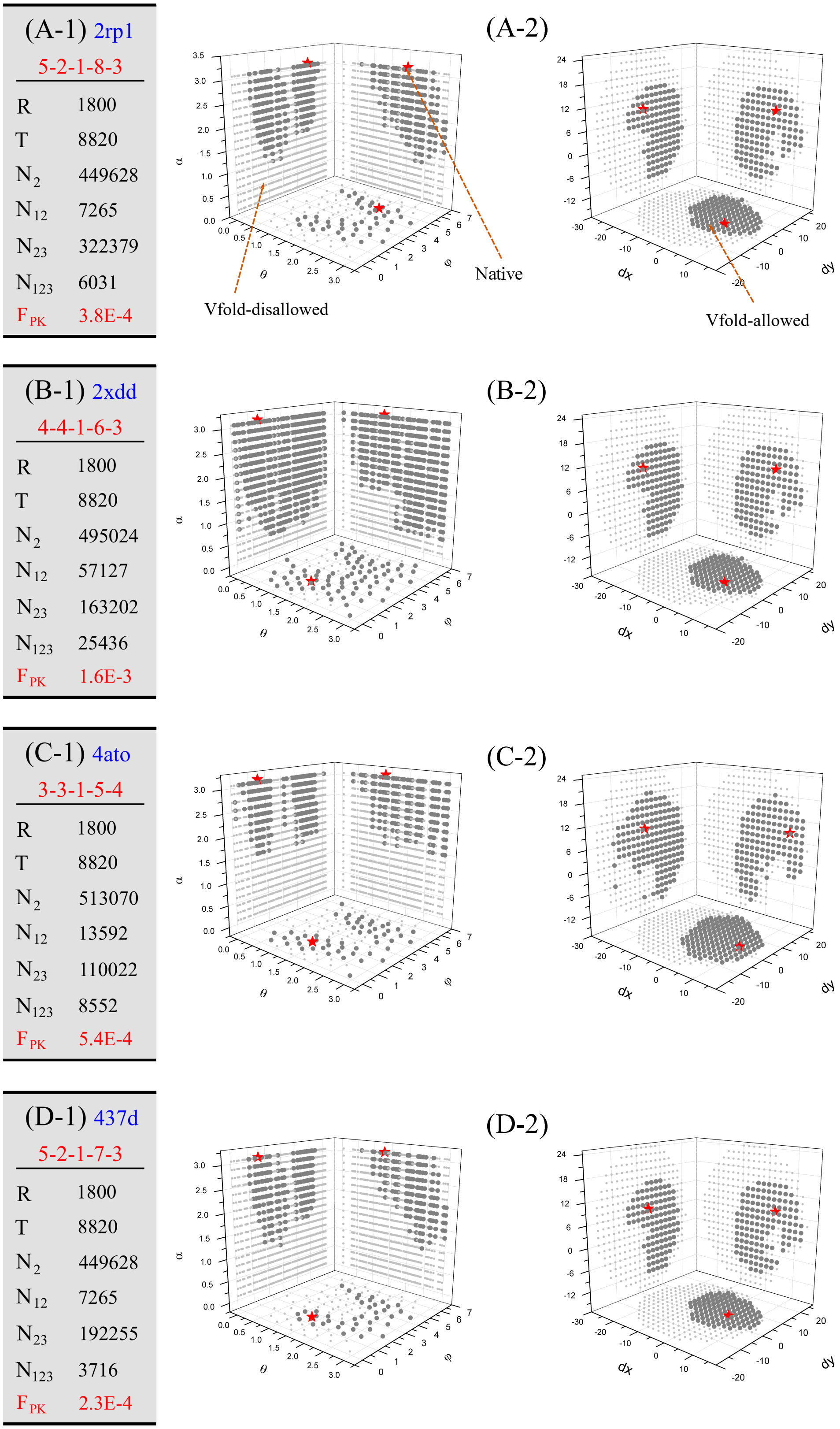

Figure S9: The quantification of the conformational sampling and the projections of the allowed (large dark gray) and disallowed (small light gray) helix configurations on six planes (three for translation, and three for rotation) for (A) 2rp1, (B) 2xdd, (C) 4ato, and (D) 437d, respectively.

(A-1) 4rmo

5-3-2-9-5

R	1800
T_{HLH}	4913
N_2	496876
N_{12}	7301
N_{23}	369671
N_{123}	7300
F_{PK}	8.3E-4

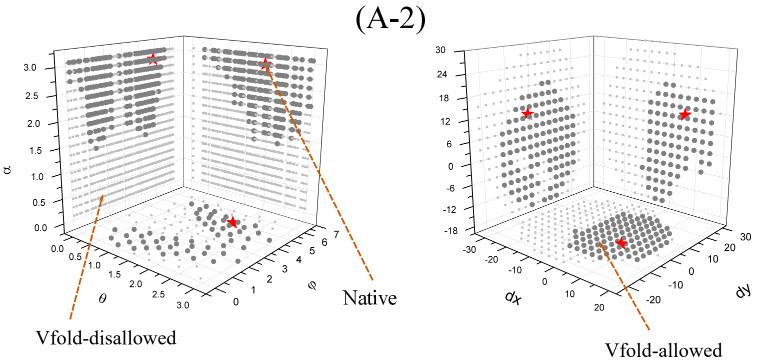

(B-1) 1kaj

5-3-2-8-4

R	1800
T	4913
N_2	501806
N_{12}	14142
N_{23}	271895
N_{123}	11965
F_{PK}	1.4E-3

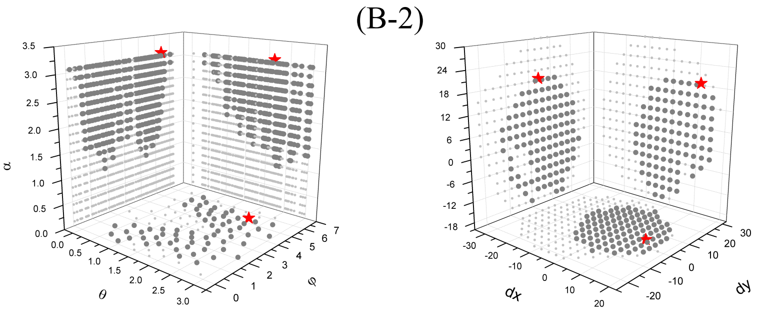

(C-1) 2m58

3-3-3-7-4

R	1800
T	3840
N_2	671440
N_{12}	22471
N_{23}	418253
N_{123}	20981
F_{PK}	3.0E-3

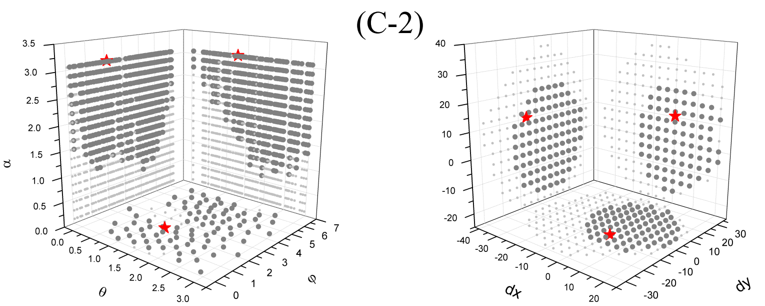

(D-1) 2m8k

5-6-0-5-12

R	1800
T	3840
N_2	181248
N_{12}	24519
N_{23}	10144
N_{123}	1096
F_{PK}	2.6E-5

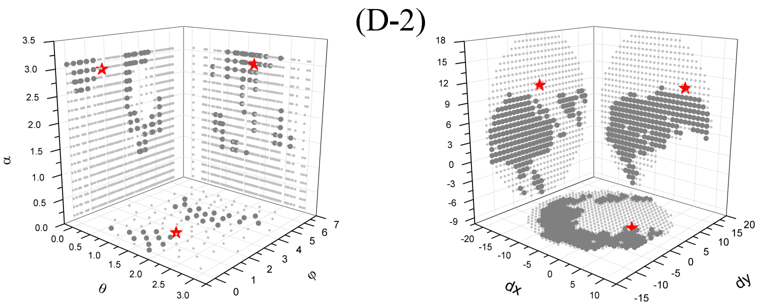

Figure S10: The quantification of the conformational sampling and the projections of the allowed (large dark gray) and disallowed (small light gray) helix configurations on six planes (three for translation, and three for rotation) for (A) 4rmo, (B) 1kaj, (C) 2m58, and (D) 2m8k, respectively.

Table S1: The sequence and 2D structure defined from the corresponding references for the cases of PK motif.

PDB	sequence/2D structure (in dot-bracket format)	Ref.
1a60	CCCCUUUUCCGAGGGUCAUCGGA<br>[[[....(((((]]]...)))))	[1]
1e95	GCGGCCAGCUCCAGGCCGCCAAACAAUAUGGAGC<br>((((((.[[[[[[)))))).........]]]]]]	[2]
1hvu	UUCCGUUUUCAGUCGGGAAAAACUGAA<br>[[[[[..((((((]]]]]...))))))	[3]
1ymo	GGGCUGUUUUUCUCGCUGACUUUCAGCCCCAAACAAAAAAGUCAGC<br>[[[[[[........(((((((((]]]]]]........)))))))))	[4]
2n6q	AACCUUCACCAAUUAGGUUCAAAUAAGUGGU<br>[[[[...((((((((]]]]....))))))))	[5]
2tpk	UGACCAGCUAUGAGGUCAUACAUCGUCAUAGC<br>[[[[[.(((((((]]]]].......)))))))	[6]
4p5j	CCCCUCUUCCGAGGGUCAUCGGAA<br>[[[...((((((]]]...))))))	[7]
2m8k	GGUUUCUUUUUAGUGAUUUUUCCAAACCCCUUUGUGCAAAAAUCAUU<br>[[[[[......((((((((((((]]]]].....).).))))))))))	[8]
2ap0	AGUGGcGCCGACCACUUAAAAACAACGG<br>(((((..[[[.))))).........]]]	[9]
1kpd	GGCGCAGUGGGCUAGCGCCACUCAAAGGCCC<br>[[[[[...(((((.]]]]].......)))))	[10]
1rnk	GGCGCAGUGGGCUAGCGCCACUCAAAAGGCCCA<br>[[[[[..((((((.]]]]]........))))))	[11]
1yg4	AGUGGcGCCGACCACUUAAAAACACCGG<br>(((((..[[[.))))).........]]]	[12]
2a43	GCGGCACCGUCCGCUCAAACAAACGG<br>((((..[[[.)))).........]]]	[13]
2rp1	UCCGGUcGACUCCGGAGAAACAAAGUC<br>(((((..[[[.)))))........]]]	[14]
2xdd	AGGUGAUUUGCUACCUUUAAGUGCA<br>((((....[[[.))))......]]]	[15]
4ato	GGUGUAACCUUACCGUAGUAGGU<br>[[[...((((.]]].....))))	[16]
437d	CGCGGCACCgucCGCGGAACAAACGG<br>(((((..[[[.))))).......]]]	[17]
4rmo	ACCACUGACCGAUAUGUGGUAUAUAAAUGGUCGG<br>(((((...[[[[[..))))).........]]]]]	[18]
1kaj	GGCGCAGUGGGCUAGCGCCACUCAAAAGCCC<br>((((....[[[[...))))........]]]]	[19]
2m58	GAGACGCCAGUCACUCAGAUAUCCUGG<br>[[[...((((...]]].......))))	[20]

Table S2: The sequence and 2D structure defined from the corresponding references for the cases of KISS motif.

PDB	sequence/2D structure (in dot-bracket format)	Ref.
1e8o	GGGCCGGGCGCGGUGGCGCGCGCCUGUAGUCCCAGCUACUCGGGAGGCUC<br>((((((((((((..[[[.)))))))....((((.]]..]..)))))))))	[21]
3skl	GGCUUAUACAGGGUAGCAUAAUGGGCUACUGACCCCGCCUUCAAACCUAUUUGGAGACUAUAAGUC<br>(((((((((..((((((.....[[))))))[.....)](((((((]]..)))))))..))))))))	[22]
3ds7	GGACAUACAAUCGCGUGGAUAUGGCACGCAAGAUCCCGCCGGGCACCGUAAAUGUCCGACUAUGUCC<br>((((((((..(.(((((.....[[))))).)[.....)]((((((]].....))))))..)))))))	[23]
3ivn	GGCCAGUAUAACCUCAAUGAUAUGGUUUGAGGGUGUCUACCAGGAACCGUAAAAUCCUGACUACUGGUC<br>(((((((((..((((((......[[.))))))[.....)](((((.]]......)))))..))))))))	[24]
4lx5	GGCUUCAUAUAAUCCGAAUGAUAUGGUUUCGGAGCUUCCACCAAGAGCCUUAAACUCUUGAUUAUGAAGUC<br>((((((((((..((((((......[[.))))))[.....)]((((((]].....))))))..)))))))))	[25]
4wfl	GCGGGGAGGUAGCGGUGCCCUGUACCUGCAAUCCGCUCUAGCAGGGC<br>((((((....[[[[[..))))...(((((...]]]]]...)))))))	[26]
4uyk	GGCCGGGGGGUUCGGCGUCCCCUGUAACCGGAAACCGCCGAUAUGCCGGGGCC<br>(((((((((...[[[[[.)))))....((((....]]]]].....))))))))	[27]

Table S3: The sequence and 2D structure extracted by RNApdbee for the cases shown in Fig. S4(C-2).

PDB	sequence/2D structure
3MR8	GCGCCGGCCAACUCCGUGCCAGCAGCCGCGGUAAUACGGAGGGCGC<br>(((((\[\[\[...(((((.....((.\]\]\])).......))))))))))
3DF1	GCACCGGCUAACUCCGUGCCAGCAGCCGCGGUAAUACGGAGGGUGC<br>(((((\[\[\[...(((((.....((.\]\]\])).......))))))))))
4B3T	GCGCCGGCCAACUCCGUGCCAGCAGCCGCGGUAAUACGGAGGGCGC<br>(((((\[\[\[...(((((.....((.\]\]\])).......))))))))))
4KJ6	GCACCGGCUAACUCCGUGCCAGCAGCCGCGGUAAUACGGAGGGUGC<br>(((((\[\[\[...(((((.....((.\]\]\])).......))))))))))
3JYV	UUGGAGGCAAGUCUGGUGCCAGCAGCCGCGGUAAUUCCAGCUCCAA<br>..(((\[\[\[....((((.....((.\]\]\])).......)))).)))..
5AJ3	GAGUUGGUAAAUCUCGUGCCAGCCACCGCGGUCAUACGAUUAACCC<br>(.(((\[\[\[.....(((.....((.\]\]\])).......)))..))).)
3J59	GCACCGGCUAACUCCGUGCCAGCAGCCGCGGUAAUACGGAGGGUGC<br>(((((\[\[\[...(((((.....((.\]\]\])).......))))))))))
3J5J	GCACCGGCUAACUCCGUGCCAGCAGCCGCGGUAAUACGGAGGGUGC<br>(((((.\[\[...(((((.....((.\]\].)).......))))))))))
3O30	UUGGAGGGCAAGUCUGGUGCCAGCAGCCGCGGUAAUUCCAGCUCCAA<br>(((((\[\[......((((.....((..\]\])).......)))).)))))
4KZY	UUGGAGGGCAAGUCUGGUCGCAGCAGCCGCGGUAAUUCCAGCUCCAA<br>((((.\[\[......((((.....((..\]\])).......))))..))))
3J43	GGGCUGGGCAAGGCCGGUGGCAGCCGCCGCGGUAAUACCGGCGGCCC<br>(((((\[\[.....(((((.....(...\]\].).......))))))))))
4D5L	UUGGAGGGCAAGUCUGGUGCCAGCAGCCGCGGUAAUUCCAGCUCCAA<br>((((.\[.......(.((.....((...\])).......)).)..))))
3JAM	CUGGAGGGCAAGUCUGGUGCCAGCAGCCGCGGUAAUUCCAGCUCCAG<br>(((.(\[\[....(.((((.....((..\]\])).......)))))).)))
4BPP	UUGGAGGGCAAGUCAUGGUGCCAGCAGCCGCGGUAAUUCCAGCUCCAA<br>(((((\[\[....(.(.(((.....((..\]\])).......))))))))))

Table S4: The sequence and 2D structure extracted by RNApdbee for the cases shown in Fig. S4(D-2).

PDB	sequence/2D structure
3J3E	GAACGCAGCAAACUGUGCGUCAUCGUGUGAACUGCAGGACACAUGAACAUCGACAUUUU<br>((((((.[[.....))).....(.((((.....]]..........))))..)...)))
4A1C	GAACGCAGCGAAAUGCGAUACGCAAUGCGAAUUGCAGAACCGCGAGUCAUCAGAUCUUU<br>((((((.[[.....))).....(.((((.....]].........).)))..)...)))
3J3F	GAACGCAGCUAGCUGCGAGAAUUAAUGUGAAUUGCAGGACACAUUGAUCAUCGACACUUC<br>.((.((.[[.....))......(.(((......]]............)))..)...)).
4BYP	GAACGCAGCGAAAUGCGAUACGUAAUGUGAAUUGCAGAAUUCCGUGAAUCAUCGAAUCUUU<br>((((((.[[.....))).....(.((((.....]]...........).)))..)...)))
3EZX	GAACGCAGCAAAGUGCGAUAAGUGGUAUCAAUUGCAGAAUCAUUUCAUUGCCCAAUCUUU<br>((((((.[[.....)))....(((((((...(.]].).........).)))).).).)))
3J0Y	GGACGUGCUAAUCUGCGAUAAGCGUCGGUAAGGUGAUAUGAACCGUUAUAACCGGCGAUUUCC<br>((.(((.[..[[[.))).......((((....(((]]]..].)))........)))).....))
3J50	GGACGUGCUAAUCUGCGAUAAGCGUCGGUAAGGUGAUAUGAACCGUUAUAACCGGCGAUUUCC<br>((((((.[..[[[.))).....((((((...(((]]]..].)))........))))))...)))
3J51	GGACGUGCUAAUCUGCGAUAAGCGUCGGUAAGGUGAUAUGAACCGUUAUAACCGGCGAUUUCC<br>((((((.[...[[.))).....((((((...(((]]...].)))........))))))...)))
3J5E	GGACGUGCUAAUCUGCGAUAAGCGUCGGUAAGGUGAUAUGAACCGUUAUAACCGGCGAUUUCC<br>((((((.[..[[[.)))....((((((((..(..]]]..]...)......))))))).).)))
3J3W	GGACGGGACGAACACCGAUAUGCUUCGGGGAGCUGUAAGCAAGCUUUGAUCCGGAGAUUUCC<br>(.((((..[[....))).....((((((...(((.]..]..)))......))))))...).)
3JQ4	GGACGCGAUUACCUGCGAAAAGCCCCGACGAGCUGGAGAUACGCUUUGACUCGGGGAUGUCC<br>(((((..[...[[..)).....((((((...((.]]...]..))......))))))...)))
2VHM	GGACGUGCUAAUCUGCGAUAAGCGUCGGUAAGGUGAUAUGAACCGUUAUAACCGGCGAUUUCC<br>(..(((......[.))).......((..(...((.]........))........)..)).......)
3BBO	GGGCGUAUUAAUCGACGAAAUGCUUCGGGGAGUUGAAAAUAAGCAGAGAUCCGGAGAUUCCC<br>..(.(.......[..)........(((.....((]......))........))).....)..
4UY8	GGACGUGCUAAUCUGCGAUAAGCGUCGGUAAGGUGAUAUGAACCGUUAUAACCGGCGAUUUCC<br>((((((.[..[[[.)))....(((((((...(((]]]..].)))........))))))).).)))
3BBX	GGACGUGCUAAUCUGCGAUAAGCGUCGGUAAGGUGAUAUGAACCGUUAUAACCGGCGAUUUCC<br>((((((.[..[[[.))).....((((((...(((]]]..].)))........))))))...)))
5ADY	GGACGUGCUAAUCUGCGAUAAGCGUCGGUAAGGUGAUAUGAACCGUUAUAACCGGCGAUUUCC<br>((((((.[...[[.)))....(((((((...(((]]...].)))........))))))).).)))
2V47	GGACGUGGCUACCUGCGAUAAGCCAGGGGGAGCCGGUAGCGGGCGUGGAUCCCUGGAUGUCC<br>((((((.[...[[.)))....(((((((...((.]]...]..))......))))))).).)))
1P9X	GGACGCGAUUACCUGCGAAAAGCCCCGACGAGCUGGAGAUACGCUUUGACUCGGGGAUGUCC<br>((((...[...[[...).....((((((...((.]]...]..))......))))))...)))
2GYA	GGACGUGCUAAUCUGCGAUAAGCGUCGGUAAGGUGAUAUGAACCGUUUAACCGGCGAUUUCC<br>...(.(.[...[[.).).......((((...(((]]...].)))......))))........
1Y69	GGACGCGAUUACCUGCGAAAAGCCCCGACGAGCUGGAGAUACGCUUUGACUCGGGGAUGUCC<br>(((((..[[..[...)).....(((.((...((..]..]]..))......)).)))...)))
1Z58	GGACGCGAUUACCUGCGAAAAGCCCCGACGAGCUGGAGAUACGCUUUGACUCGGGGAUGUCC<br>(((.(......[[..)...........(...(..]].......)......).........)))
2B9P	GGACGUGGCUACCUGCGAUAAGCCAGGGGGAGCCGGUAGCGGGCGUGGAUCCCUGGAUGUCC<br>((.((.......[..)).....((((((...(((]......)))......))))))....))

Table S5: The sequence and 2D structure used for the benchmark test.

PDB	sequence/2D structure
1a60	GGGAGCUCAACUCUCCCCCCCUUUUCCGAGGGUCAUCGGAACCA<br>(((((.......)))))(((...[[[[[[)))...]]]]]]...
3mr8	GCGCCGGCCAACUCCGUGCCAGCAGCCGCGGUAAUACGGAGGGCGC<br>(((((([[[...(((((.....((.]]])).......))))))))))
5kh8	GGCGAUGGUGUUCGCCAUAAACGCUCUUCGGAGCUAAUGACACCUAC<br>((((..[[[[[[))))......((((....))))....]]]]]]...
6fz0	AGGCGCAUUUGAACUGUAUUGUACGCCUUGCA-GCAAAAGUACUAAAAA<br>((((..............[[[[[[))))((..-.))...]]]].]]...
4ena	GGGCGAUGAGGCCCGCCCAAACUGCCCUGAAAAGGGCUGAUGGCCUCUACUG<br>.((((..[[[[[[))))......(((((....)))))....]]]]]].....
4rge	CCUGUGAGGGUCCUAAGCCCCUAAUUCAGAAGGGAAA-UUUUUAAUGAAGCCACAGG<br>((((((.(((.[[[..{{)))...((((..]]].((.-.))....))))}}))))))
2m58	GAAGAAAGGGCUUCGGCCACUCAAACUACAGAGACGCCAGUCACUCAGAUAUCCUGGU<br>(((((((.(((....)))))))))).....(((...[[[[...))).......]]]].
2miy	GCUUGGUGCUUAGCUUCUUUCACCAAGCAUAUUACACGCGGAUAACCGCCAAAGGAGAA<br>((((((((.....[[[[[[.)))))))).........((((....))))..]]]]]]..
3bbx	GGACGUGCUAAUCUGCGAUAAGCGUCGGUAAGGUGAUAUGAACCGUUAUAACCGGCGAUUUCC<br>((((((.[..[[[.))).....((((((...(((]]]..].))).......))))))...)))
5ktj	ACUCGUUUGAGCGAGUAUAAACAGUUGGUUAGGCUCAAAGCGGAGAGCAG-UCUGCUCUCGUCCAA<br>((((([[[[[[)))))........((((....(]]]]]].).((((((((-.))))))))..))))
2n8v	GGAACAGCUGUACUGGGCAGUUACAGCAGUCGUAUGGUAACACAUGCGGCGUUCCGAAAUACCAUGCCUG<br>(((((.((((((..[[[[[..)))))).((((((((......))))))))))))))).........]]]]].
5kpy	GGACACUGAUGAUCGCGUGGAUAUGGCACGCAUUGAAUUGUUGGACACCGUAAAUGUCCUAACACGUGUCC<br>((((((......(.(((((.....[[))))))..........(((((]].....)))))......))))))
5tpy	GGGUCAGGCCGGCGAAAGUCGCCACAGUUUGGGGAAAGCUGUGCAGCCUGUAACCCCCCCACGAAAGUGGG<br>....((((((((....)))).((((((..[[[[...))))))..)))))...]]]](((((....)))))
5d5l	GAAGGCCGAAAGGUCUUCCACGACGAUACUUAUUUCCUUUGAUCGUCGUUAUUACUGGCUUCGGCCACAAAGGAGA<br>(((((((....))))))).((((((((.......[[[[[..)))))))).......((((....))))..]]]]]..
4jf2	GGAACCGCGAAAGCGGUUCCACGACGAUACUUAUUUCCUUUGAUCGUCGUUAUUACUGGCUUCGGCCACAAAGGAGA<br>((((((((....))))))))((((((((.......[[[[[..))))))))......((((....))))..]]]]]..
4p5j	AGCUCGCCAGUUAGCGAGGUCUGUCUCGACACGACAGAUAAUCGGGUGCAACUCCCGCCCCUCUUCCGAGGGUCAUCGGAACCA<br>..(((((..[...)))))(((((((.......)))))))...((((..]....))))(((...[[[[[[)))...]]]]]]...
3t4b	CCUCCCGGGAGAGCCGCUAAGGGGGAAACUCUAUGCGGUACUGCCUGAUAGGGUGCUUGCGAGUGCCCCGGGAGGUCUCGUAGA<br>((((((((((.(((((...((((....))))..))))).))((((....))))((..[[[[[.))))))))))).]]]]]...
5fjc	GGCUUAUCAAGAGAGGGGGAGUGACUGGCGCGAAGAACCCCGGCAACCAGAAAUGGUGCCAAUUCCUGCAGCGGAAACGUUGAA<br>((((((((....(.(((...(((.[.[[))).......))))(((..(((....))).)))....]].](((((....)))))..<br>AGAUGAGCCG<br>.)))))))).
4rzd	GAGCAACUUAGGAUUUUAGGCUCCCCGGCGUGU-CGAACCAUGCCGGGCCAAACCCAUAGGGCUGGCGGUCCCUGUGCGGUCAA<br>((((......[[[[[[...))))(((((((((.-.....)))))))))....((((((((((........))))))).)))..]<br>AAUUCAUCCGCCGGAG<br>]]]]]..........
6mj0	AAGUUCUCGAUCUUUAAAAUCGUUAGCUCGCCAGUUAGCGAGGUCUGCGAAAGCAGAUAAUCGGGUGCAACUCCCGCCCUUUCU<br>(((....[[[[))))....]]]]...((((((..[...)))))((((((....))))))...((((..]....))))(((....[<br>CCGAGGGUCAUCGGAAC<br>[[[.))).)..]]]]..
4frn	GGGCUAAAAGCAUGGUGGGAAAGUGACGUGUAAUUCGUCCACAUUACUUGAUACGGUUAUACUCCGAAUGCCACCUAGCCCAAA<br>((((((.......(((((....(.((((.......))))){.((..[[[[)).(((......}))))....))))))))))...<br>GUAGAGCAAGGAGACUCA<br>...(((]]]]....))).
4wfl	GGCCGUGCUAAGCGGGGAGGUAGCGGUGCCCUGUACCUGCAAUCCGCUCUAGCAGGGCCGAAUCCCUUCUCGAGGUUCGGUAAC<br>.(((((((...((((((....[[[[[..))))...(((((...]]]]]...)))))))......((((((...((((((....)<br>GAAUCGACAGAAGGUGCACGGUC<br>)))))...)))))).))))))).

References

[1] Kolk MH, et al. NMR structure of a classical pseudoknot: interplay of single- and double-stranded RNA. (1998). *Science*, **280**: 434-438.

[2] Michiels PJA, et al. Solution structure of the pseudoknot of SRV-1 RNA, involved in ribosomal frameshifting. (2001). *J Mol Biol*, **300**: 1109-1123.

[3] Jaeger J, et al. The structure of HIV-1 reverse transcriptase complexed with an RNA pseudoknot inhibitor. (1998). *EMBO J*, **17**: 4535-4542.

[4] Theimer CA, et al. Structure of the human telomerase RNA pseudoknot reveals conserved tertiary interactions essential for function. (2005). *Molecular Cell*, **17**: 671-682.

[5] Cash DD, et al. Structure and folding of the Tetrahymena telomerase RNA pseudoknot. (2017). *Nucleic Acids Res*, **45**: 482-495.

[6] Holland JA, et al. An examination of coaxial stacking of helical stems in a pseudoknot motif: The gene 32 messenger RNA pseudoknot of bacteriophage T2. (1999). *RNA*, **5**: 257-271.

[7] Colussi TM, et al. The structural basis of transfer RNA mimicry and conformational plasticity by a viral RNA. (2014). *Nature*, **511**: 366-370.

[8] Cash DD, et al. Pyrimidine motif triple helix in the Kluyveromyces lactis telomerase RNA pseudoknot is essential for function in vivo. (2013). *Proc Natl Acad Sci USA*, **110**: 10970-10975.

[9] Cornish PV, et al. The global structures of a wild-type and poorly functional plant luteoviral mRNA pseudoknot are essentially identical. (2006). *RNA*, **12**: 1959-1969.

[10] Kang H, et al. A mutant RNA pseudoknot that promotes ribosomal frameshifting in mouse mammary tumor virus. (1997). *Nucleic Acids Res*, **25**: 1943-1949.

[11] Shen LX, et al. The Structure of an RNA pseudoknot that causes efficient frameshifting in mouse mammary tumor virus. (1995). *J Mol Biol*, **247**: 963-978.

[12] Cornish PV, et al. A loop 2 cytidine-stem 1 minor groove interaction as a positive determinant for pseudoknot-stimulated -1 ribosomal frameshifting. (2005). *Proc Natl Acad Sci USA*, **102**: 12694-12699.

[13] Pallan PS, et al. Crystal structure of a luteoviral RNA pseudoknot and model for a minimal ribosomal frameshifting motif. (2005). *Biochemistry*, **44**: 11315-11322.

[14] Nixon PL, et al. Solution structure of a luteoviral P1-P2 frameshifting mRNA pseudoknot. (2002). *J Mol Biol*, **322**: 621-633.

[15] Fineran PC, et al. The phage abortive infection system, ToxIN, functions as a protein-RNA toxin-antitoxin pair. (2009). *Proc Natl Acad Sci USA*, **106**: 894-899.

[16] Short FL, et al. Selectivity and self-assembly in the control of a bacterial toxin by an antitoxic noncoding RNA pseudoknot. (2012). *Proc Natl Acad Sci USA*, **111**: E241-E249.

[17] Su L, et al. Minor groove RNA triplex in the crystal structure of a ribosomal frameshifting viral pseudoknot. (1999). *Nature Struct Biol*, **6**: 285-292.

[18] Rao F, et al. Co-evolution of quaternary organization and novel RNA tertiary interactions revealed in the crystal structure of a bacterial protein-RNA toxin-antitoxin system. (2015). *Nucleic Acids Res*, **43**: 9529-9540.

[19] Kang H, et al. Conformation of a Non-frameshifting RNA pseudoknot from mouse mammary tumor virus. (1996). *J Mol Biol*, **259**: 135-147.

[20] Carlomagno T, et al. Structure principles of RNA catalysis in a 2’-5’ Lariat-forming ribozyme. (2013). *J Am Chem Soc*, **135**: 4403-4411.

[21] Weichenrieder O, et al. Structure and assembly of the Alu domain of the mammalian signal recognition particle. (2000). *Nature*, **408**: 167-173.

[22] Pikovskaya O,et al. Structural principles of nucleoside selectivity in a 2’-deoxyguanosine riboswitch. (2011). *Nat Chem Biol*, **7**: 748-755.

[23] Edwards AL, Batey RT. A structural basis for the recognition of 2’-deoxyguanosine by the purine riboswitch. (2009). *J Mol Biol*, **385**: 938-948.

[24] Delfosse V, et al. Riboswitch structure: an internal residue mimicking the purine ligand. (2009). *Nucleic Acids Res*, **38**: 2057-2068.

[25] Robinson CJ, et al. Modular riboswitch toolsets for synthetic genetic control in diverse bacterial species. (2014). *J Am Chem Soc*, **136**: 10615-10624.

[26] Kempf G, et al. Structure of the complete bacterial SRP Alu domain. (2014). *Nucleic Acids Res*, **42**: 12284-12294.

[27] Bousset L, et al. Crystal structure of a signal recognition particle Alu domain in the elongation arrest conformation. (2014). *RNA*, **20**: 1955-1962.
